# Enhancing Team Strategies and Tools to Enhance Performance and Patient Safety Performance Through Medical Movies, Massive Open Online Courses, and 3D Virtual Simulation–Based Interprofessional Education: Mixed Methods Double-Blind Quasi-Experimental Study

**DOI:** 10.2196/67001

**Published:** 2025-09-08

**Authors:** Khuansiri Narajeenron, Thippaya Chintakovid, Phanupong Phutrakool, Jiraphan Ritsamdang, Atthaphon Viriyopase, Khrongwong Musikatavorn, Sirikanyawan Srikasem, Noppawan Boonbumrong, Chulaluk Jaipang, Sunisa Seephom, Kanyarat Susantitapong, Nuntaree Chaichanawongsaroj, Sararas Khongwirotphan, Sawitree Suayod, Porntiwa Sunpawut, Tippayaporn Pavavimol, Pataraporn Kheawwan, Chanya Thanomlikhit, Suwimon Rojnawee, Thititip Tippayamontri, Kawin Dhanakoses, Nattanun Chanchaochai, Sujinat Jitwiriyanont, Natasha Sahapattana, Sirhavich Khowinthaseth, Ora-In Chu, Tewarit Sarachana, Navaporn Worasilchai, ER-VIPE study group (Emergency Room – Virtual Interprofessional Education)

**Affiliations:** 1 Department of Emergency Medicine Faculty of Medicine, Chulalongkorn University King Chulalongkorn Memorial Hospital, The Thai Red Cross Society Bangkok Thailand; 2 Department of Library Science, Faculty of Arts Chulalongkorn University Bangkok Thailand; 3 Chula Data Management Center, Faculty of Medicine Chulalongkorn University Bangkok Thailand; 4 Department of Pharmacy Practice Faculty of Pharmaceutical Sciences Chulalongkorn University Bangkok Thailand; 5 Department of Emergency Medicine Faculty of Medicine, Chulalongkorn University King Chulalongkorn Memorial Hospital, The Thai Red Cross Society Bangkok Thailand; 6 Adult and Gerontological Nursing Department Srisavarindhira Thai Red Cross Institute of Nursing Bangkok Thailand; 7 Department of Pharmacy Section King Chulalongkorn Memorial Hospital, , The Thai Red Cross Society Bangkok Thailand; 8 Department of Transfusion Medicine and Clinical Microbiology Faculty of Allied Health Sciences Chulalongkorn University Bangkok Thailand; 9 Department of Radiological Technology and Medical Physics Faculty of Allied Health Sciences Chulalongkorn University Bangkok Thailand; 10 Department of Pediatric Nursing Srisavarindhira Thai Red Cross Institute of Nursing Bangkok Thailand; 11 Department of Mass Communication Faculty of Communication Arts Chulalongkorn University Bangkok Thailand; 12 Faculty of Nursing Chulalongkorn University Bangkok Thailand; 13 Nursing Professional Development Center, Department of Nursing King Chulalongkorn Memorial Hospital, The Thai Red Cross Society Bangkok Thailand; 14 Department of Radiological Technology and Medical Physics, and Cancer and Aging Research Unit Faculty of Allied Health Sciences Chulalongkorn University Bangkok Thailand; 15 Department of Architecture, Faculty of Architecture Chulalongkorn University Bangkok Thailand; 16 Department of Linguistics and Center of Excellence in Southeast Asian Linguistics, Faculty of Arts Chulalongkorn University Bangkok Thailand; 17 Faculty of Medicine Chulalongkorn University Bangkok Thailand; 18 Department of Clinical Chemistry, Medical Technology Program, and the Autism Research and Innovation Center of Excellence (ChulaACE) Faculty of Allied Health Sciences Chulalongkorn University Bangkok Thailand; 19 Department of Transfusion Medicine and Clinical Microbiology, Medical Technology Program, and the Medical Mycology Diagnostic Center of Excellence (MedMyCoE) Faculty of Allied Health Sciences Chulalongkorn University Bangkok Thailand; 20 Chulalongkorn University Bangkok Thailand

**Keywords:** interprofessional education, TeamSTEPPS, Team Strategies and Tools to Enhance Performance and Patient Safety, virtual reality simulation, medical movie, massive open online courses, co-debriefing, computer-based, virtual reality, mixed methods, emergency medicine

## Abstract

**Background:**

The interprofessional educational curriculum for patient and personnel safety is of critical importance, especially in the context of the COVID-19 pandemic, to prepare junior multiprofessional teams for emergency settings.

**Objective:**

This study aimed to evaluate the effectiveness of an innovative interprofessional educational curriculum that integrated medical movies, massive open online courses (MOOCs), and 3D computer–based or virtual reality (VR) simulation-based interprofessional education (SimBIE) with team co-debriefing to enhance interprofessional collaboration and team performance using Team Strategies and Tools to Enhance Performance and Patient Safety (TeamSTEPPS). This study addressed 3 key questions. First, it aimed to examine the impact of 3D computer–based and VR SimBIE with co-debriefing following medical movies and MOOCs on improving TeamSTEPPS competencies compared to 3D computer–based SimBIE without preparation or debriefing. Second, it explored learners’ perceptions of TeamSTEPPS effectiveness using the New World Kirkpatrick model. Third, it assessed differences in Simulator Sickness Questionnaire scores between the 3D and VR SimBIE formats.

**Methods:**

This mixed methods, prospective, double-blinded (raters and statistician) quasi-experimental study was conducted at a university hospital from August 2022 to September 2023 and involved 87 clinical undergraduate students from various disciplines, including medicine, nursing, pharmacy, radiology, and medical technology. Students were divided into 3 arms: arm A (control) received 3D computer–based SimBIE without debriefing; arm B received a medical movie, MOOCs, 3D computer–based SimBIE, and co-debriefing; and arm C received the same as arm B but with VR-based SimBIE. The validated Modified TeamSTEPPS Team Performance Observation Tool was used to measure team performance. A 60- to 90-minute focus group discussion with semistructured interview questions, based on the New World Kirkpatrick model and aligned with the TeamSTEPPS framework, was conducted with subsequent thematic analysis.

**Results:**

In total, 87 participants were enrolled in this study. TeamSTEPPS’ performance was significantly higher (*P*<.001) in arms B and C, which received comprehensive training with medical videos, MOOCs, and 3D computer–based or VR-based SimBIE with co-debriefing, compared to the control group. Additionally, the analysis of focus group discussions based on Kirkpatrick’s model levels 1 to 3 indicated positive effects on satisfaction, engagement, knowledge, skills, attitude, and confidence, enriched by game practice and debriefing sessions. Simulator Sickness Questionnaire scores increased significantly in arm C (VR; Δ=21.0; *P*=.001) but not in arm B (computer; Δ=5.3; *P*=.44).

**Conclusions:**

Integrating TeamSTEPPS with medical movies, MOOCs, 3D computer–based or VR-based SimBIE training, and co-debriefing effectively improved interprofessional collaboration and team performance in emergency medicine. We recommend adopting these novel instructional designs as standard preparation of undergraduate health care professionals for better clinical practice to improve patient and personnel safety and patient outcomes.

## Introduction

Preventable medical errors are a challenging public health problem and commonly occur in health care, posing a substantial threat to patient safety [1,2]. Effective communication and teamwork among physicians, staff, and patients are critical in emergency care settings to minimize medical errors and promote a culture of safety [3-6]. The World Health Organization (WHO) Global Patient Safety Action Plan was adopted at the World Health Assembly, providing a 10-year roadmap and actions to enhance patient safety and reduce the burden of patient harm due to unsafe health care [7]. In Thailand, the Healthcare Accreditation Institute (HAI), a public organization, follows WHO guidelines and is responsible for setting hospital quality standards and certifying health care services. This organization also promotes a patient and personnel safety (2P Safety) curriculum to prepare future health care leaders [2]. However, existing patient safety curriculum often lacks experiential learning opportunities across professions and are typically delivered outside clinical settings, limiting their effectiveness in real-world applications, as noted by Piyawan Limpanyalert, the director of the HAI (public organization), Thailand (face-to-face meeting, May 26, 2023).

Efficient interprofessional education (IPE) is an approach to teaching and learning, in which students from 2 or more professional backgrounds learn about topics from each other, enabling effective collaboration, teamwork, and communication and improving health outcomes [8-10]. IPE can be delivered through various methods, including e-learning and simulation [11]. One example is Team Strategies and Tools to Enhance Performance and Patient Safety (TeamSTEPPS), an evidence-based framework designed to enhance teamwork, communication, and efficiency in health care settings, ultimately improving patient safety and care quality [12,13].

Simulation has been shown to improve teamwork and communication skills in health care professional students [14]. Mahmood et al [15] incorporated TeamSTEPPS into interprofessional simulation–based training with debriefing, a structured reflection process conducted after an experience to analyze and integrate lessons learned [16], which has also been shown to enhance teamwork and communication. However, physical simulations face scalability challenges. In contrast, 3D computer–based or virtual reality (VR) simulations, powered by advanced technology, provide greater accessibility for interprofessional students both on-site and remotely. Additionally, 3D computer–based or VR simulations offer standardized training, real-time feedback, cost-effectiveness, and the ability to customize complex scenarios through programming, making them a versatile and scalable alternative [17].

Learning through simulation can enhance learning outcomes by preparing multiprofessional students with a foundational understanding of TeamSTEPPS principles, terminology, and a common language [18]. Adequate preparation using a flipped classroom approach has been shown to enhance learning effectiveness and teaching quality by helping learners build confidence and increase their ability to apply teamwork strategies more effectively, particularly in simulated crisis situations [19,20]. To support this, our project integrated movies [21] and massive open online courses (MOOCs) combined with formative quizzes as scalable, accessible, and cost-effective preparatory tools [22,23]. These resources provided students with essential knowledge and attitudes prior to engaging in simulation-based interprofessional education (SimBIE), which focuses on clinical emergency scenarios [24].

This study aimed to evaluate the effectiveness of an IPE curriculum integrating medical movies, MOOCs, and 3D computer–based or VR-based SimBIE with co-debriefing, guided by Kolb’s experiential learning theory, which consists of 4 stages: concrete experience, reflective observation, abstract conceptualization, and active experimentation [25]. This study addressed 3 key questions. First, it aimed to examine the impact of 3D computer–based and VR SimBIE with co-debriefing following medical movies and MOOCs on improving TeamSTEPPS competencies compared to 3D computer–based SimBIE without preparation or debriefing. Second, it explored learners’ perceptions of TeamSTEPPS effectiveness using the New World Kirkpatrick model. Third, it assessed differences in Simulator Sickness Questionnaire (SSQ) scores between the 3D and VR SimBIE formats.

## Methods

### Design and Setting

**Figure 1:**
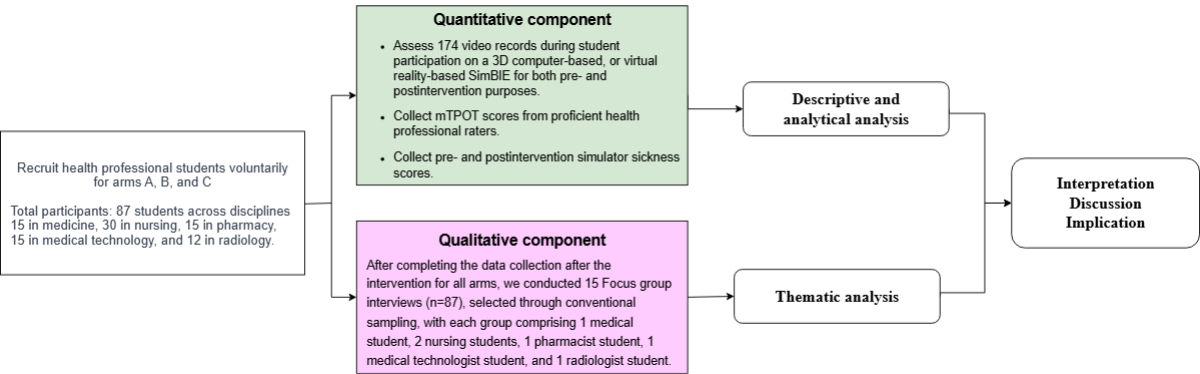
The mixed methods concurrent triangulation research design. mTPOT: Modified TeamSTEPPS Team Performance Observation Tool; SimBIE: simulation-based interprofessional education.

We conducted a mixed methods, double-blind (raters and statistician), prospective quasi-experimental study (Figure 1) at a single university hospital between August 2022 and September 2023. Students were assigned to three groups, as outlined in the CONSORT (Consolidated Standards of Reporting Trials) flow diagram and CONSORT checklist (Multimedia Appendix 1):

1. Control arm A (n=29): Engaged in 3D computer–based SimBIE without prior preparation (eg, movies or MOOCs on TeamSTEPPS) or debriefing.
2. Experimental arm B (n=29): Participated in a comprehensive program, including a medical movie, MOOCs, 3D computer–based SimBIE, and a debriefing session.
3. Experimental arm C (n=29): Received the same interventions as arm B, but with 3D VR SimBIE instead of the computer–based version.

This study was conducted as action research, with the aim of comparing a conventional, real-world teaching model—which mirrors common limitations in typical emergency education settings—represented by group A, with a novel IPE multimodal instructional design that integrated preparatory learning (eg, MOOC and medical movie–based education) and structured co-debriefing. The differences in instructional content and structure between groups were intentional and reflect current educational practices versus enhanced, IPE multimodal learning approaches. Rather than isolating a single intervention variable under strictly controlled conditions, the study was designed to inform practical improvements in teaching strategies that support teamwork competencies in health care education.

### Participants

A total of 147 volunteers registered to participate in the study. The volunteers were from five health care disciplines: (1) fifth- or sixth-year medical students, (2) fifth- or sixth-year pharmacy students, (3) fourth-year medical technology students, (4) third- or fourth-year radiological technology students, and (5) third- or fourth-year nursing students. Following institutional review board approval, the study was announced through multiple communication channels, including verbal announcements, Line, and posters, targeting students from the relevant disciplines. Interested participants enrolled via a QR code linked to a Google Forms, which also provided the contact information of the principal investigator (KN) for further inquiries. To ensure minimal disruption to academic schedules, participants selected time slots that did not interfere with their regular coursework.

Eligible volunteers of the study were healthy individuals aged from 18 to 25 years and did not meet the following exclusion criteria: (1) missed 2 scheduled appointments, (2) violated preparation guidelines of the experiment, and (3) had a psychological disease diagnosed by a medical professional, scoring ≥9 on the Patient Health Questionnaire-9 [26,27]. The exclusion criteria related to psychological diseases and Patient Health Questionnaire-9 were implemented due to a concurrently conducted study requiring this specification.

Of the 147 volunteers, 87 met the eligibility criteria and were enrolled in the study. Due to the predetermined structure of clinical rotation schedules amid the COVID-19 pandemic, random allocation of participants was not practical. Consequently, a stratified convenience sampling strategy was used to allocate students into 3 groups. This helps ensure comparability between groups in terms of key baseline characteristics, including profession, age, level of education, and prior clinical exposure. To accommodate participants’ schedules, all volunteers indicated their availability and selected time slots that did not conflict with their academic coursework. To minimize group bias, a 2-way blinding process was implemented: participants were unaware of their assigned group, and research administrators allocated participants to unlabeled time slots without access to individual identities.

### Intervention

#### Medical Movie

The participants engaged in a structured learning activity by watching “Emergency Love,” a 75-minute medical film depicting interprofessional collaboration (IPC) in the emergency department (ED). Developed by a multidisciplinary team of experts—including specialists in medicine, psychology, communication arts, literature, and aviation—the film also incorporated insights from airline captains with expertise in crew resource management, ensuring a high-quality screenplay and production. This cinematic resource was used to impart essential insights into TeamSTEPPS principles, situational awareness, IPC, teamwork dynamics, communication strategies, leadership attributes, team clinical reasoning for diagnosis, decision-making, ethical dilemmas, professionalism standards, and management of stress and fatigue. The movie featured both positive and negative role models, providing a comprehensive exploration of critical aspects relevant to emergency room scenarios within the context of health care education.

#### Massive Open Online Course

The participants completed a 1.45-hour MOOC titled “Inter-Professional Collaboration for Patient Safety.” The course consisted of 7 sections covering nontechnical skills, such as TeamSTEPPS, communication for IPC, ethical principles for IPC, team critical thinking for diagnosis, coping strategies, Interprofessional Education Collaborative core competencies, and 2P Safety goal. Each section included a quiz, and there were 2 assessments (pre- and posttest). All participants in arms B and C were required to complete the course and obtain certification by passing both the quizzes and the posttest.

#### 3D Virtual Clinical Simulation Scenario

The 30- to 60-minute scenario involved a 70-year-old patient with his wife presenting to the ED with acute dyspnea and febrile illness, alongside a history of chronic obstructive pulmonary disease, hypertension, diabetes mellitus, and ceftriaxone allergy. Recently discharged from the intensive care unit, his vital signs and laboratory results indicated critical hyperkalemia and COVID-19 pneumonia with acute respiratory failure. Participants engaged in the Emergency Room-Virtual Interprofessional Education (ER-VIPE) platform, an innovative VR platform for medical IPE in Thailand. This nonopen educational resource simulation was designed to reinforce TeamSTEPPS principles introduced through medical movies and MOOCs and to apply these insights in practice to enhance 2P Safety [28].

The learning objectives focused on applying TeamSTEPPS strategies to enhance 2P Safety, emphasizing effective communication, leadership, mutual support, situational monitoring, and structured teamwork within an interprofessional health care environment.

Each scenario involved 6 participants (5 professional roles) communicating via computer microphones (non-VR) or VR headsets, using direct conversation, individual calls, and a group call function (limited to 3 uses per scenario). Participants could not pass objects directly and were limited to holding 1 item at a time. They could boost teammates’ morale—up to 5 times, excluding themselves—by pressing a “plus” button to restore the Health Point bar, a visual indicator of emotional resilience that decreased with stress or delayed actions. This feature simulated cognitive load and reinforced mutual support behaviors aligned with TeamSTEPPS.

**Figure 2:**
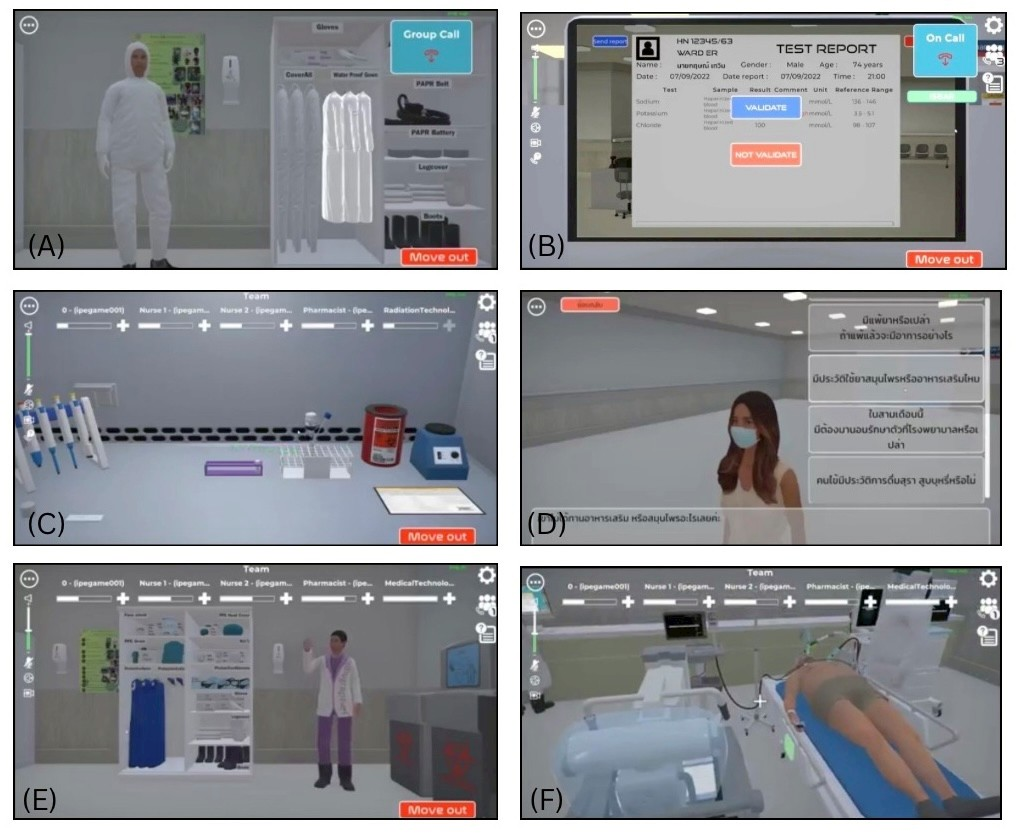
Communication and interaction features across 6 professional roles in 3D computer-based SimBIE. (A) Group call discussion among physicians wearing personal protective equipment. (B) A medical technologist informing a physician of a critical potassium result via an individual call. (C) Both individual and group call functionalities across disciplines, shown from a medical technologist’s perspective during laboratory work. (D) Medication reconciliation, with a pharmacist collecting a patient’s medication history from a family member to assess allergies and current prescriptions. (E) A radiologic technologist donning personal protective equipment before performing a portable chest x-ray. (F) Real-time communication in a shared 3D computer–based space, including patient wristband identification and collaborative bed rail adjustments to support fall prevention and mutual support. SimBIE: simulation-based interprofessional education.

Additional functionalities included wristband-based patient identification and 1-time laboratory test ordering. Figure 2 highlights key collaborative and communication features, along with limitations, within the gameplay environment. It showcases participant engagement across 6 professional roles in 3D computer–based scenarios as part of 3D computer–based SimBIE. Image A depicts group call discussions among physicians in personal protective equipment, while image B shows a medical technologist informing a physician of a critical potassium result via an individual call. Image C illustrates both individual and group call functionalities across disciplines, viewed from a medical technologist’s perspective during laboratory work. Image D demonstrates medication reconciliation, with a pharmacist collecting a patient’s medication history from a family member to assess allergies and current prescriptions. Image E portrays a radiologic technologist donning personal protective equipment before performing a portable chest x-ray. Image F represents real-time communication in a shared 3D computer–based space, showcasing collaborative features such as patient wristband identification and collaborative bed rail adjustments to enhance fall prevention and mutual support. This structured simulation immerses participants in a dynamic, interprofessional environment, fostering the application of TeamSTEPPS strategies in future real-world clinical scenarios.

#### Co-Debriefing Process

The co-debriefers were multiprofessional staff facilitators, not simulation participants. Their role was to observe and document key learner behaviors—both strengths and areas for improvement—during SimBIE gameplay and facilitate debriefing using the Gather-Analyze-Summarize (GAS) model to guide discussions. This approach aimed to enhance learning by addressing gaps based on TeamSTEPPS objectives and developing actionable strategies for future similar situations, rather than actively participating as players. To maximize the benefits of cofacilitation and ensure active participation from all professions, we used a structured co-debriefing approach. Each profession was assigned specific TeamSTEPPS topics to lead, promoting shared ownership of the learning process. A designated debriefing leader coordinated predebriefing, debriefing, and postdebriefing sessions [29]. To ensure clear goals and role assignments and to allocate tasks equitably within the allotted 1-hour time frame, we used proactive planning. During debriefing sessions, cofacilitators fostered a respectful, open, and supportive environment through active listening and negotiation. To reinforce learning, we incorporated an after-action review phase following each debriefing. Debriefing was structured according to the GAS model [30], comprising three phases: (1) gather: creation of a safe environment and collection of participants’ impressions; (2) analyze: co-debriefers’ in-depth examination of performance, using the plus-delta approach, advocacy inquiries to promote critical self-reflection, and recognizing commendable practices as well as potential improvement; and (3) summarize: integration of insights to reinforce understanding of the learning objectives and their practical implications. A team of 2-5 experienced instructors facilitated the debriefing and provided personalized feedback and addressed overlooked issues to refine the participants’ competencies.

### Assessment Tools

#### Overview

In this study, we adopted the TeamSTEPPS framework to evaluate IPC among health care students [31]. This framework focuses on 5 essential areas: team structure, communication, leadership, situation monitoring, and mutual support, all of which are crucial for effective patient care. A validated Modified TeamSTEPPS Team Performance Observation Tool (mTPOT) [32], assessing behaviors across these categories, was used to measure student performances in the 3D computer–based or VR-based SimBIE.

#### Development and Content Validity of the mTPOT

The original authors of the Team Performance Observation Tool (TPOT) granted permission to modify and use the instrument for research and educational purposes. The TPOT was translated and validated against a Thai version [32]. Under the Kane-Messick framework, modifications to the TPOT were made to increase the validity of the scoring process [33]. For each item in the TPOT, behavioral anchors were defined a priori*.* Anchors were directly related to the events in the clinical simulation scenario, and separate behavioral anchors were defined for each profession. Anchoring was also performed for rating scales (eg, score 1=none of the defined tasks were done and score 5=completed all of the defined tasks). Anchoring ensured a stronger representation of each item to measure the intended construct and avoided rater bias stemming from the vagueness of a simple 1-5 rating scale. For example, the behavioral anchors defined for “using check-backs to verify information” for the physician and nursing professions were the (1) patient name, (2) drug name, (3) drug dose, (4) drug route, (5) drug rate, and (6) critical K level. A rating of excellent would be given for successful verification of all 6 items, and a rating of very poor would be given for 0 of 6 items verified by use of check-backs. Items were removed for professions that did not have a specific behavioral anchor suitable for the simulation.

Content validation for the behavioral anchors was achieved through discussion, group consensus, and revision under the judgment of 3 medical experts in crisis management, simulation education, and nontechnical skills (consisting of 2 anesthesiologists and 1 emergency medicine physician). Modifications, rewording, and omissions to behavioral anchors were made until a final version of the mTPOT was reached. The experts were asked to rate each anchor with a score of 1-4 to indicate their agreement with the anchor’s ability to accurately measure the construct of the item in the original TPOT. All items achieved an item-level content validation index, and the scale-level content validation index scores were >0.8, signifying consensus within the group that the behavioral anchors possessed a high level of content validity [34].

#### An Example of the Validated mTPOT

To ensure the applicability of mTPOT within the VR or computer-based simulation context, specific items were adapted. For instance, item 1.4 involving patients and their families as part of the team was modified to assess team behaviors in obtaining informed consent and incorporating patient or family input during decision-making. The adapted assessment criteria included (1) explicitly engaging patients in decision-making—such as obtaining informed consent for procedures (eg, intubation or tracheostomy) and (2) assigning team members to gather patient history—including drug allergies, current medications, and past hospitalizations—from either the patient or their family.

The scoring system for this adapted item was structured as follows: 5=excellent: team members obtain informed consent and conduct a complete history-taking, actively involving the patient or family in decision-making; 4=good: the team attempts to obtain consent and history but does so at an inappropriate time or with incomplete information; 3=fair: both consent and history-taking are attempted, but poorly executed; 2=poor: only 1 of the 2 behaviors (consent or history-taking) is performed; and 1=inadequate: neither consent nor history-taking is performed.

This approach ensures that the mTPOT remains relevant in a 3D computer–based or VR-based SimBIE, assessing team communication and decision-making with direct patient-family interaction. TeamSTEPPS scoring (mTPOT) for physicians is provided in Multimedia Appendix 2 as an example, with data available for all 5 professions across 6 roles. However, the complete mTPOT data and assessment tools are proprietary and not publicly available.

#### Trained Rater Methods

In our study, the TeamSTEPPS raters belonged to 5 professional groups (12 raters), including 2 emergency doctors, 4 nurses, 2 pharmacists, 2 medical technologists, and 2 radiological technologists. These raters were trained to assess the TeamSTEPPS assessment form. They analyzed the participants’ performance by reviewing video recordings, remaining unaware of the participants’ group allocation (control or experimental groups) and without knowledge of the pre- and postintervention conditions, making them blinded assessors.

A threat to the validity of observational performance assessments was the subjective variation arising from the raters themselves, such as rater error and bias. Rater training was necessary to develop the correct skills and attitudes involved in accurately evaluating desired competencies; the following three methods were used to this end [35-37]: (1) a 10-minute rater error training session discussed common rater biases and aimed toward developing raters’ understanding and metacognition of common tendencies toward bias; (2) a 40-minute performance dimension training session focused on the mTPOT criteria, providing clear definitions and examples (anchors) to illustrate each item on the assessment scale; and (3) a 60-minute frame of reference rater training group discussion was conducted to review results and address any discrepancies, with particular focus on professions improving interrater agreement. Raters were provided with 3 videos showing examples of behavior relating to items being evaluated. The videos either showed high quality (matching all of the evaluated criteria), moderate quality (matching most of the evaluated criteria), or low quality (matching few of the evaluated criteria) behavior. Next, a multiple-choice pretest was given to each rater with a textual example of a scenario describing the desired behavior, with raters asked to use the mTPOT rating scale to choose the appropriate level of desired behavior displayed. Subsequently, each textual scenario was discussed in depth, with particular attention to discrepant scenarios, in which ratings differed among raters from the same profession. The raters discussed their thought processes behind their ratings and received feedback.

#### mTPOT Assessment

A double-blind method was used, blinding both assessors and a statistician. A research assistant (AV) was responsible for obscuring sensor data, such as the date and time within the recording screen, of 174 video clips. The video reviews captured profession-specific perspectives, as shown in Figure 2, for both 3D computer–based and VR-based SimBIE, ensuring that raters assessed participants within their roles while maintaining evaluation consistency and contextual relevance. The research assistant (AV) assigned study codes to each clip to indicate if it belonged to the pre- or postfinal assessment phase and if it was from the control or intervention arms. Only the research assistant (AV) had knowledge of the coding system. In the prefinal assessment, mTPOT performance was measured at baseline during the first round of gameplay. In the postfinal assessment, mTPOT performance was measured during the second round of gameplay.

Notably, the standardized scenario remained the same for both the first and second rounds of gameplay. To minimize technical issues during the simulation, the participants were allowed to familiarize themselves with the game controller beforehand.

The research assistant (AV) distributed the video links by email, along with instructions and a submission form for mTPOT scores, to 12 assessors from various health care professions. Each assessor was responsible for evaluating 30 clips, except for the radiological technologists, who evaluated 24 clips. The evaluation process spanned 3 months.

The statistician (PP), to whom the study codes were not revealed, received the assessment results for analysis without knowing whether the clips were from the pre- or postfinal assessment phase or to which group (control or intervention) they belonged. The research assistant (AV) then separated the analysis results by study code to identify which clips were in the pre- or postfinal assessment phase without revealing the group allocation.

#### Motion Sickness and Simulator Sickness Assessment

Although 3D computer–based or VR-based SimBIE provided valuable opportunities for experiential learning, they posed limitations for certain learners, particularly those susceptible to simulator sickness or cybersickness. These conditions could serve as confounding variables, influencing both the learning process and the development of teamwork skills among participants. Therefore, our team evaluated these factors by examining the participants’ histories of motion sickness through the Motion Sickness Susceptibility Questionnaire, with scores ranging from 0 to 54 [38], and by assessing simulator sickness before and after the initial game session using the SSQ, with scores ranging from 0 to 235.62 [39]. Notably, the initial SSQ measurement was done after the participants had familiarized themselves with the game controller. This approach allowed for a comprehensive understanding and accurate assessment of the impact on learning outcomes.

#### Qualitative Focus Group Method

After participation in a 3D computer–based or VR simulation, the participants in each group were asked to attend a focus group led by TC, a female PhD researcher with about 10-15 years of experience in quantitative and qualitative research in studying user interaction with new technology. Each focus group consisted of students from 5 disciplines, including 1 medical student, 2 nursing students, 1 pharmacy student, 1 medical technology student, and 1 radiological technology student. Semistructured interview questions were developed on the basis of levels 1 and 2 of the New World Kirkpatrick model [40] and aligned with the TeamSTEPPS framework. Preliminary interview sessions were conducted with a few groups of students joining the 3D computer–based simulation at the early phase of the simulation design and development. The goal of these sessions was to determine if the interview questions were easy to understand and if the order of questions was appropriate. The questions were then refined before being used in this study. The interviewer asked for participants’ informed consent prior to joining the focus group session, which lasted approximately 60 to 90 minutes. During the focus group, unclear answers were clarified immediately by asking the participants to explain further. The interviews were audio-recorded, and all spoken words were then transcribed. Additionally, to support the qualitative components of our study, we have completed and attached a COREQ (Consolidated Criteria for Reporting Qualitative Research) checklist for the interview and focus group components, included in Multimedia Appendix 3 [41].

### Statistical Analysis

#### Overview

STATA/MP statistical software release 15 (StataCorp LLC) was used to perform the data analysis. Weighted κ statistics were used to assess the interrater agreement for tool development. Cronbach α was used to measure internal consistency and reliability. Descriptive statistics were used to summarize the data. Continuous variables were presented as the mean and SD along with the median and IQR. Categorical variables were summarized as frequencies and percentages. The chi-square test, the Fisher exact test, the Wilcoxon Mann-Whitney *U* test, and the Kruskal-Wallis *H* test with Dunn post hoc test were used to assess the differences in baseline characteristics between treatment groups. Generalized estimating equations were used to analyze longitudinal changes and assess differences in improvement scores between groups, adjusting for health care disciplines as covariates. A significance level of *P*<.05 was accepted as indicating statistical significance. Rater blinding was applied during evaluation, while statistician blinding was implemented during data analysis to minimize potential bias. The statistician remained unaware of group assignments throughout the analysis, and the principal investigator (KN) unblinded the groups only after the full analysis was completed.

#### Sample Size Calculation

The sample size was determined using a power analysis for multivariate analysis of variance with repeated measures, conducted in G*Power (version 3.1.9.4; Heinrich Heine University Düsseldorf). A total of 82 participants was required to detect an effect size of 0.35 with α set to .05 and 80% power. This effect size, larger than a small effect (0.2) but below a medium effect (0.5), remains clinically and practically significant in this study. To account for potential dropout, 6% (n=5) of the participants were added to each group. The total sample size was 87 participants.

#### Qualitative Data Analysis

Two researchers, one (TC) with approximately 10-15 years of quantitative and qualitative research experience and the other with approximately 5 years of qualitative research experience, were interviewers of the focus group sessions. One of the researchers performed 2 rounds of transcription: an initial verbatim transcription using Transkriptor (Transkriptor, Inc), a transcription software converting recorded interview files to text files; and a second round of checking the accuracy of the transcribed texts by reviewing the interview transcripts against the recorded audio files.

The research used a thematic analysis [42] of the transcribed interviews to understand the students’ learning experience with the simulations. Guided by the New World Kirkpatrick model and TeamSTEPPS principles, the 2 researchers independently gave a first pass through the interview transcripts of the first focus group and assigned codes focusing on the students’ learning experiences, including knowledge and skills acquired, feelings toward the teaching methods, confidence levels, and feedback for instruction improvement. After this initial code assignment, the 2 researchers met to compare and discuss the codes and developed a codebook, documented in a spreadsheet program, for subsequent analysis. Following the codebook development, the 2 researchers separately assigned codes for the rest of the transcribed interviews, then read the codes together and discussed any codes assigned differently. Discrepancies in coding were refined and finalized on the basis of the discussion. By examining patterns found across the codes, initial themes were identified according to levels 1 and 2 of the New World Kirkpatrick model. Another iteration refined the themes and assigned the codes concerning “game practice” and “debriefing” additional themes on the basis of level 3 of the New World Kirkpatrick model. To enhance the depth and rigor of the analysis, another round of qualitative analysis was implemented, drawing upon the 5 domains of the TeamSTEPPS principles.

### Ethical Considerations

Ethics approval was granted by the institutional review board of the Faculty of Medicine, Chulalongkorn University, in accordance with the Declaration of Helsinki (COA: 1085/2022). All participants gave written informed consent and were assured that their data would be kept confidential. They were also informed that participation was voluntary and that they could withdraw from the study at any time without penalty. To support their time and contribution, each participant received compensation of US $30 as approved by the institutional review board.

## Results

### Demographics and Enrollment

**Figure 3:**
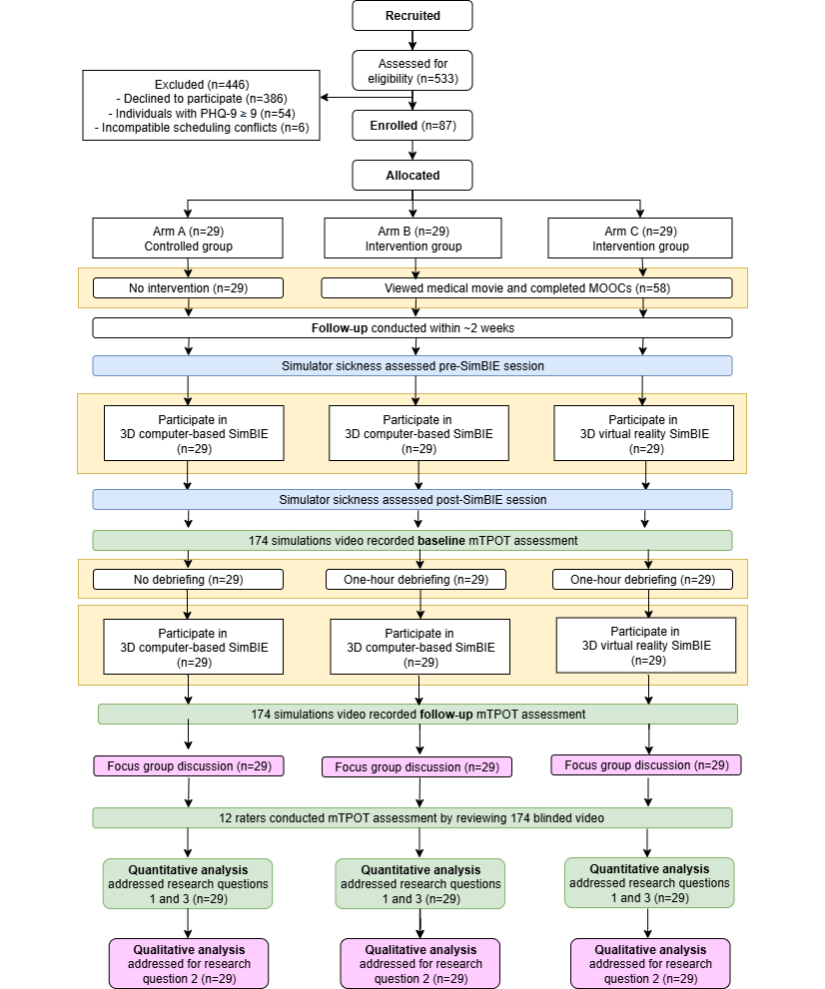
CONSORT flowchart of the study. MOOC: massive open online course; mTPOT: Modified TeamSTEPPS Team Performance Observation Tool; PHQ-9: Patient Health Questionnaire-9; SimBIE: simulation-based interprofessional education.

A total of 533 participants were recruited and assessed for eligibility. After excluding 446 participants, the final study sample included 87 students. The sample comprised 15 medical students, 30 nursing students, 15 pharmacy students, 15 medical technology students, and 12 radiological technology students (Figure 3), meeting the initially calculated sample size requirement of 87 participants.

The majority of the participants were female (60/87, 69%). The mean age of the cohort was 21.87 (SD 1.16) years. Among the medical students, 12/15 (80%) were in their fifth year of study, whereas 3/15 (20%) were in their sixth year. Nursing students were distributed across the third (13/30, 43%) and fourth years (17/30, 57%) of their program. Pharmacy students were enrolled in the fourth (2/15, 13%), fifth (6/15, 40%), and sixth years (7/15, 47%) of their respective curricula. All radiological technology and medical technology students were in their fourth year of study (Multimedia Appendix 4). Notably, most health care professional curricula under Thailand’s academic system span 4 years, with the exception of medical and pharmacy programs, which require 6 years for completion. All participants were clinical students. An analysis of demographic characteristics revealed no significant differences between the groups (*P>*.05), as presented in Table 1.

**Table 1:** Demographics and baseline by treatment arm.

Factors			Total (N=87)		Arm A (n=29)		Arm B (n=29)		Arm C (n=29)		*P* value	
**Sex, n (%)**												>.99^a^
	Female	60 (69)		20 (69)		20 (69)		20 (69)				
	Male	27 (31)		9 (31)		9 (31)		9 (31)				
**Age (years)**												
	Mean (SD)	21.87 (1.16)		21.83 (1.04)		21.86 (1.03)		21.93 (1.41)		—^b^		
	Median (IQR)	22 (21-22)		22 (21-22)		22 (21-22)		21 (21-22)		.91^c^		
**Academic year, n (%)**												.70^a^
	3	13 (15)		4 (14)		7 (24)		2 (7)				
	4	46 (53)		16 (55)		13 (45)		17 (59)				
	5	18 (21)		6 (21)		5 (17)		7 (24)				
	6	10 (11)		3 (10)		4 (14)		3 (10)				
**Academic grade**												
	Mean (SD)	3.22 (0.38)		3.20 (0.40)		3.23 (0.43)		3.22 (0.31)		—		
	Median (IQR)	3.24 (2.98-3.50)		3.25 (2.93-3.53)		3.23 (3.03-3.50)		3.24 (3.00-3.48)		.94^c^		
**PHQ-9^d^ score**												
	Mean (SD)	3.72 (3.04)		3.62 (2.38)		3.48 (2.98)		4.07 (3.70)		—		
	Median (IQR)	4 (1-6)		4 (2-5)		3 (0-5)		4 (1-6)		.88^c^		

^a^Fisher exact test.

^b^Not tested.

^c^Kruskal-Wallis *H* test.

^d^PHQ-9: Patient Health Questionnaire-9.

### Mean Changes in mTPOT Scores for Each Intervention Arm

The mean range of the minimum and maximum mTPOT scores across the 5 domains was 1-5. The overall mean mTPOT scores at baseline were 2.04 (95% CI 1.91-2.17) for arm A, 2.75 (95% CI 2.62-2.89) for arm B, and 2.88 (95% CI 2.74-3.01) for arm C. The mean mTPOT score at baseline for arm A was significantly different from those of both arm B and arm C (*P*<.001; Multimedia Appendix 5).

**Figure 4:**
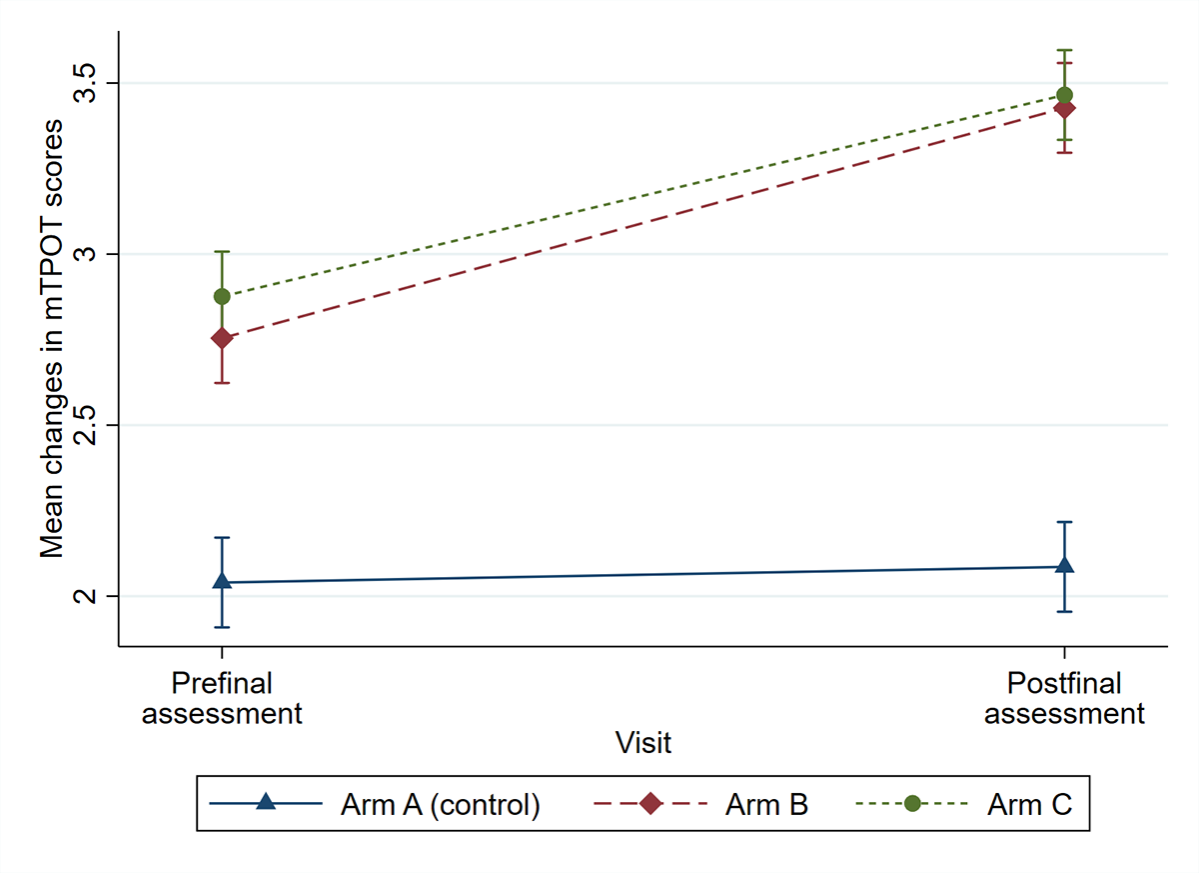
Illustration of the substantial improvement in the mean mTPOT score change between pre- and postfinal assessments in arm B and arm C compared to the control group (arm A). mTPOT: Modified TeamSTEPPS Team Performance Observation Tool.

**Figure 5:**
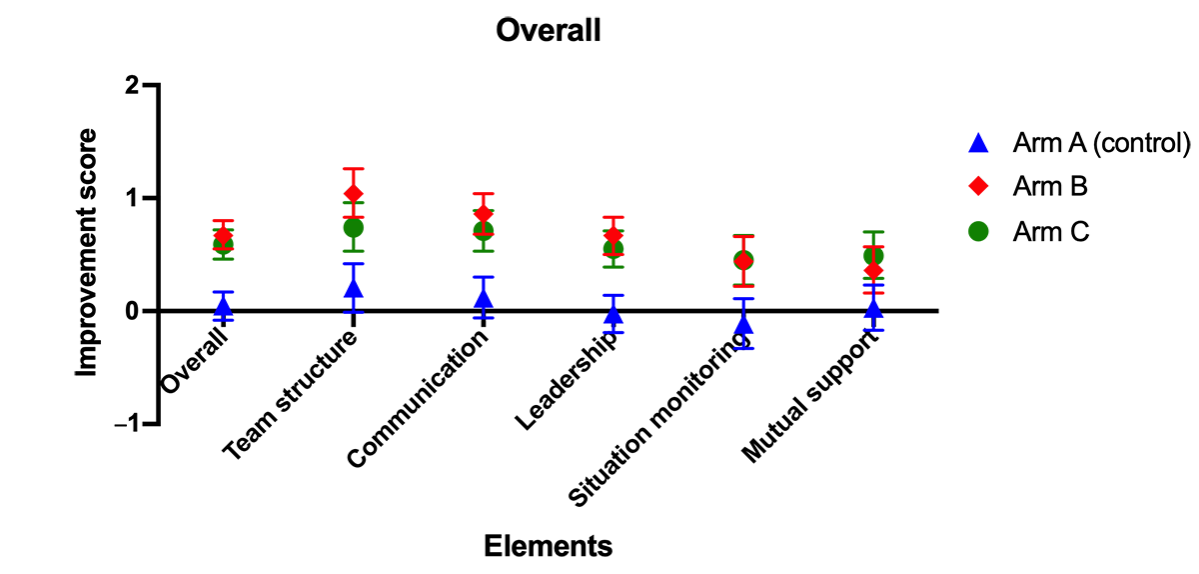
Overall improvement in mTPOT scores across TeamSTEPPS domains. mTPOT: Modified TeamSTEPPS Team Performance Observation Tool; TeamSTEPPS: Team Strategies and Tools to Enhance Performance and Patient Safety.

Significant changes from baseline total scores were observed in both arm B and arm C following the intervention, with improvements of 0.67 (95% CI 0.55-0.80; *P*<.001) and 0.59 (95% CI 0.46-0.72; *P*<.001), respectively. Additionally, all TeamSTEPPS domain scores showed significant improvement in both arm B and arm C (*P*<.001). In contrast, no significant changes were observed in the control group (arm A) for either the overall mTPOT scores or within the 5 TeamSTEPPS domains (Figures 4 and 5 and Table 2).

When comparing the interventions, the mean improvement in scores for arm B was 0.63 (95% CI 0.45-0.81; *P*<.001), which was significantly higher than that of the control group. Similarly, the mean improvement in scores for arm C was 0.54 (95% CI 0.36-0.72; *P*<.001), which was also significantly higher than that in the control group. However, no significant differences were observed between arm B and arm C (*P*=.59; Table 2 and Figures 4 and 5).

In key TeamSTEPPS domains, significant increases were observed in the team structure, communication, and leadership scores in arm B, with improvements of 1.04 (95% CI 0.83-1.26), 0.86 (95% CI 0.68-1.04), and 0.67 (95% CI 0.50-0.83), respectively (*P*<.001). Similarly, in arm C, improvements in team structure, communication, and leadership scores were 0.74 (95% CI 0.53-0.96), 0.71 (95% CI 0.53-0.89), and 0.55 (95% CI 0.39-0.71), respectively (*P*<.001). Although there were increases in situation monitoring and mutual support scores, the improvements were modest, ranging from 0.36 to 0.49 (*P*<.001; Table 2 and Figure 5).

**Table 2:** Changes in mean Modified TeamSTEPPS Team Performance Observation Tool scores before and after final assessment^a^.

Domain		Arm				Pairwise comparisons		
		A, mean (95% CI)	B, mean (95% CI)	C, mean (95% CI)	Arm B versus A, mean (95% CI)		Arm C versus A, mean (95% CI)	Arm C versus B, mean (95% CI)
**Overall**								
	Prefinal assessment	2.04 (1.91 to 2.17)	2.75 (2.62 to 2.89)	2.88 (2.74 to 3.01)	—^b^		—	—
	Postfinal assessment	2.09 (1.95 to 2.22)	3.43 (3.30 to 3.56)	3.47 (3.33 to 3.60)	—		—	—
	Difference	0.05 (–0.08 to 0.17)	0.67 (0.55 to 0.80)	0.59 (0.46 to 0.72)	0.63 (0.45 to 0.81)		0.54 (0.36 to 0.72)	–0.08 (–0.26 to 0.10)
	*P* value	.48	<.001^c^	<.001^c^	<.001^c^		<.001^c^	.36
**Team structure**								
	Prefinal assessment	1.54 (1.29 to 1.79)	2.92 (2.68 to 3.17)	3.35 (3.10 to 3.59)	—		—	—
	Postfinal assessment	1.75 (1.50 to 1.99)	3.97 (3.72 to 4.21)	4.09 (3.84 to 4.34)	—		—	—
	Difference	0.21 (–0.01 to 0.42)	1.04 (0.83 to 1.26)	0.74 (0.53 to 0.96)	0.83 (0.53 to 1.14)		0.53 (0.23 to 0.84)	–0.30 (–0.60 to 0.01)
	*P* value	.06	<.001^c^	<.001^c^	<.001^c^		.001^c^	.06
**Communication**								
	Prefinal assessment	2.21 (2.04 to 2.38)	2.78 (2.61 to 2.94)	2.97 (2.80 to 3.13)	—		—	—
	Postfinal assessment	2.33 (2.17 to 2.50)	3.63 (3.47 to 3.80)	3.68 (3.51 to 3.85)	—		—	—
	Difference	0.12 (–0.06 to 0.30)	0.86 (0.68 to 1.04)	0.71 (0.53 to 0.89)	0.74 (0.48 to 1.00)		0.59 (0.33 to 0.85)	–0.15 (–0.40 to 0.11)
	*P* value	.19	<.001^c^	<.001^c^	<.001^c^		<.001^c^	.27
**Leadership**								
	Prefinal assessment	2.18 (2.03 to 2.34)	2.88 (2.73 to 3.03)	2.95 (2.79 to 3.10)	—		—	—
	Postfinal assessment	2.16 (2.01 to 2.31)	3.54 (3.39 to 3.69)	3.50 (3.35 to 3.65)	—		—	—
	Difference	–0.02 (–0.19 to 0.14)	0.67 (0.50 to 0.83)	0.55 (0.39 to 0.71)	0.69 (0.46 to 0.92)		0.57 (0.34 to 0.80)	–0.11 (–0.34 to 0.12)
	*P* value	.79	<.001^c^	<.001^c^	<.001^c^		<.001^c^	.33
**Situation monitoring**								
	Prefinal assessment	2.60 (2.43 to 2.76)	2.99 (2.83 to 3.16)	2.91 (2.74 to 3.07)	—		—	—
	Postfinal assessment	2.49 (2.33 to 2.65)	3.43 (3.27 to 3.59)	3.35 (3.19 to 3.52)	—		—	—
	Difference	–0.11 (–0.33 to 0.11)	0.44 (0.22 to 0.66)	0.45 (0.23 to 0.67)	0.55 (0.23 to 0.86)		0.55 (0.24 to 0.87)	0.01 (–0.30 to 0.32)
	*P* value	.34	<.001^c^	<.001^c^	.001^c^		<.001^c^	.95
**Mutual support**								
	Prefinal assessment	1.67 (1.48 to 1.85)	2.20 (2.02 to 2.38)	2.22 (2.03 to 2.40)	—		—	—
	Postfinal assessment	1.69 (1.51 to 1.88)	2.57 (2.38 to 2.75)	2.71 (2.53 to 2.89)	—		—	—
	Difference	0.03 (–0.17 to 0.23)	0.36 (0.16 to 0.57)	0.49 (0.29 to 0.70)	0.33 (0.05 to 0.62)		0.46 (0.18 to 0.75)	0.13 (–0.16 to 0.42)
	*P* value	.78	<.001^c^	<.001^c^	.02^d^		.002^e^	.38

^a^Statistical tests: generalized estimating equations, adjusting for health care disciplines as covariates.

^b^Not tested.

^c^*P*<.001.

^d^*P*<.05.

^e^*P*<.01.

### Interrater Reliability

The overall interrater reliability across all professions exceeded 85% agreement, with weighted κ values ranging from 0.49 to 0.98, indicating good to excellent agreement beyond chance. During the prefinal assessment, the weighted κ agreement among interprofessional raters ranged from 85% to 99%, with κ statistics between 0.50 and 0.97. In the postfinal assessment, the level of agreement ranged from 87% to 99%, with weighted κ statistics between 0.49 and 0.98. Notably, of the 12 interprofessional raters, the κ statistics for the doctor and airway nurse were <0.6 in both assessments. In contrast, the circulation nurse, medical technologist, and radiological technologist consistently achieved κ statistics >0.7 in both the pre- and postfinal assessments. The κ statistics for each rater remained consistent between the 2 assessment phases (Multimedia Appendix 6).

### Analysis of Intervention Effects and Confounding Factors

After accounting for confounding factors, the Wilcoxon Mann-Whitney *U* test revealed no significant differences between arm B and arm C in the mean movie duration (mean 80.39, SD 8.39 minutes), MOOC time (mean 133.25, SD 8.91 minutes), and the number of debriefing staff (mean 5.33, SD 1.75), with all *P* values exceeding .05. The mean duration of the debriefing sessions was 54.96 (SD 12.29) minutes. A significant difference in debriefing duration was observed between arm B and arm C (median 49, IQR 45-59 vs median 63, IQR 60-69; *P*=.01). Notably, the participants in arm C, who engaged in 3D VR simulations, experienced longer debriefing sessions (Table 3).

The results indicated that the participants’ susceptibility level to motion sickness, as measured by the Motion Sickness Susceptibility Questionnaire, did not differ significantly between treatment arms (*P*=.33). The total SSQ scores at baseline were recorded following the initial team simulation and individual game practice, revealing a range from the lowest scores in arm B of 19.36 (95% CI 4.41-34.31) to the highest in arm C of 35.98 (95% CI 24.54-47.43; Table 4). Posttraining results indicated a significant increase in SSQ scores for arm C, which used a VR headset, showing the highest change in SSQ score of 21.02 (95% CI 10.67-31.37; *P*=.001). In contrast, arm B, which used computer-based games, exhibited an insignificant change in SSQ scores, with a difference of 5.28 (95% CI –8.24 to 18.80; *P*=.44). Additionally, there were no significant differences in the number of participants using antimotion sickness medication after the initial simulation between arm B and arm C (*P*=.47).

**Table 3:** Analysis of intervention effects and confounding factors.

Factors			Arm A (n=29)		Arm B		Arm C		*P* value
**Movie time (minutes)**					n=27		n=29		
	Mean (SD)	—^a^		81.19 (9.55)		79.66 (7.24)		—	
	Median (IQR)	—		83 (73-88)		81 (72-85)		.31^b^	
**MOOC^c^ time (minutes)**					n=28		n=29		
	Mean (SD)	—		133.89 (9.38)		132.62 (8.55)		—	
	Median (IQR)	—		134 (128-139.50)		132 (128-137)		.40^b^	
**Debrief staff (person)**					n=29		n=29		
	Mean (SD)	—		5.69 (1.85)		4.97 (1.59)		—	
	Median (IQR)	—		6 (4-7)		6 (5-6)		.06^b^	
**Debriefing time (minutes)**					n=28		n=21		
	Mean (SD)	—		50.93 (12.61)		60.33 (9.75)		—	
	Median (IQR)	—		49 (45.00-58.50)		63 (60-69)		.01^b,d^	
**Motion sickness score (MSSQ^e^)**			n=18		n=17		n=28		
	Mean (SD)	8.64 (7.25)		12.62 (13.32)		6.18 (7.34)		—	
	Median (IQR)	7.13 (2.57-12.90)		5.25 (1.50-20.10)		4.40 (1.06-7.69)		.33^f^	

^a^Not applicable.

^b^Wilcoxon Mann-Whitney *U* test.

^c^MOOC: massive open online course.

^d^*P*<.05.

^e^MSSQ: Motion Sickness Susceptibility Questionnaire.

^f^Kruskal-Wallis *H* test.

**Table 4:** Changes in Simulator Sickness Questionnaire score between the pre- and postfinal assessments^a^.

Factors		Arm				Pairwise comparisons		
		A, mean (95% CI)	B, mean (95% CI)	C, mean (95% CI)	Arm B versus A, mean (95% CI)		Arm C versus A, mean (95% CI)	Arm C versus B, mean (95% CI)
**Nausea**								
	Pretraining	20.32 (9.98 to 30.67)	15.15 (3.12 to 27.19)	22.70 (13.48 to 31.91)	—^b^		—	—
	Posttraining	20.32 (9.98 to 30.67)	17.96 (5.92 to 29.99)	35.20 (25.98 to 44.41)	—		—	—
	Difference	0.00 (–10.11 to 10.11)	2.81 (–8.95 to 14.56)	12.50 (3.50 to 21.50)	2.81 (–12.70 to 18.31)		12.50 (–1.03 to 26.04)	9.69 (–5.11 to 24.50)
	*P* value	>.99	.64	.007^c^	.72		.07	.20
**Oculomotor**								
	Pretraining	17.80 (7.78 to 27.82)	16.50 (4.84 to 28.15)	31.10 (22.18 to 40.03)	—		—	—
	Posttraining	20.43 (10.41 to 30.45)	24.52 (12.87 to 36.18)	52.54 (43.61 to 61.46)	—		—	—
	Difference	2.64 (–6.77 to 12.04)	8.03 (–2.92 to 18.97)	21.43 (13.06 to 29.81)	5.39 (–9.04 to 19.82)		18.80 (6.20 to 31.39)	13.41 (–0.37 to 27.19)
	*P* value	.58	.15	<.001^d^	.46		.003^c^	.06
**Disorientation**								
	Pretraining	23.60 (6.17 to 41.04)	19.65 (–0.63 to 39.93)	43.68 (28.15 to 59.21)	—		—	—
	Posttraining	28.45 (11.01 to 45.88)	20.47 (0.19 to 40.75)	64.32 (48.79 to 79.85)	—		—	—
	Difference	4.84 (–10.68 to 20.36)	0.82 (–17.23 to 18.87)	20.64 (6.82 to 34.46)	–4.02 (–27.83 to 19.78)		15.80 (–4.98 to 36.58)	19.82 (–2.91 to 42.55)
	*P* value	.54	.93	.003^c^	.74		.14	.09
**Total**								
	Pretraining	23.09 (10.24 to 35.94)	19.36 (4.41 to 34.31)	35.98 (24.54 to 47.43)	—		—	—
	Posttraining	25.69 (12.84 to 38.54)	24.64 (9.69 to 39.59)	57.00 (45.56 to 68.45)	—		—	—
	Difference	2.60 (–9.02 to 14.23)	5.28 (–8.24 to 18.80)	21.02 (10.67 to 31.37)	2.68 (–15.15 to 20.51)		18.42 (2.85 to 33.99)	15.74 (–1.29 to 32.77)
	*P* value	.66	.44	.001^c^	.77		.02^e^	.07

^a^Statistical tests: generalized estimating equations.

^b^Not tested.

^c^*P*<.01.

^d^*P*<.001.

^e^*P*<.05.

### Thematic Analysis

The codes were initially organized based on the New World Kirkpatrick model. Further analysis revealed the alignment of the focus group discussions with the TeamSTEPPS framework. Textbox 1 presents quotations demonstrating the participants’ experiences and perceptions across all domains of TeamSTEPPS. Collaborative learning methods, such as games and 3D computer–based or VR-based SimBIE scenarios, were particularly noted for their effectiveness in promoting teamwork and fostering interprofessional understanding, areas where traditional classroom settings were found lacking. Although initially met with skepticism, structured communication techniques, such as Identify, Situation, Background, Assessment, and Recommendation (ISBAR), ultimately proved valuable in practice, reducing errors and ensuring accurate information exchange. Leadership skills were notably enhanced through VR scenarios, which encouraged participants to ask questions and collaborate more effectively. Situation monitoring taught the participants the importance of assessing team readiness and the environment before communicating critical information, thereby enhancing patient safety. The value of mutual support among professionals was also highlighted, as it had a crucial role in improving patient care through collaborative efforts. Finally, the debriefing sessions reinforced the importance of careful planning and consideration, promoting a mindset focused on continuous improvement.

**Representative quotations from focus group discussions aligned with the TeamSTEPPS framework. Abbreviations denote the intervention arm, group number, and profession. Intervention arms included arms A, B, and C, and each arm consisted of 5 groups of participants. The following letters represent each profession: D for doctor, N for nurse, P for pharmacist, MT for medical technologist, and RT for radiological technologist. For instance, C5-P indicates an interview of a pharmacist in group 5 of the intervention arm C.**

**Team structure**

- “... In the classroom, we only saw theories, ... MOOCs or movies, saw visuals, and did not interact with others. When it came to the virtual situation, we spoke more with other people. It helped make ... a more complete image of teamwork” [C5-P].
- “I understand the scope of responsibility of each profession ... Knowing this makes it easy to divide the workload. For the matter of communication, ... I feel that being able to talk, meet, and get to know one another is crucial” [B5-D].

**Communication**

- “I learned to use ISBAR communication and call back a lot ... it was a recheck for myself and made sure that it was the right patient, really diagnosed this person so it didn’t cause any confusion” [B4-D].
- “It is a matter of calling back to avoid errors due to plainly speaking ... It is a recurrent process to lessen our own mistakes” [B4-N].

**Leadership**

- “... I have developed communication skills to create a team atmosphere ... I told my teammates what I observed and my thoughts about this patient because I believed that my teammates ... from various professions have studied and performed very well ... undoubtedly would have more suggestions for this patient ...” [B2-D].
- “... the game gives me more (confidence) ... We work collaboratively with nurses ... when obtaining the specimen, the nurse will inform the patient’s characteristics and gender that play a role in interpreting the findings ... We must assist one another in each profession ... Working as a team is similar to merging multiple pieces of knowledge” [B1-MT].

**Situation monitoring**

- “... (I) learned to observe the emotions and feelings of teammates to see if they were ready to receive this message ... This will cause the reception of the message to be incomplete or inaccurate, causing misunderstandings” [B4-D].
- “... Then there’s the issue of observation ... I should monitor the environment to see whether it is a good time for me to enter or not ... when I placed the image plate underneath the patient who had a needle in his body, I should pay more attention and exercise greater caution” [C3-RT].

**Mutual support**

- “... if the doctor requested something and had a specimen like this, I could search for information about the patient’s condition ... I would think about ... any other tests that could help the doctor reach a preliminary diagnosis more quickly ...” [B2-MT].
- “... The feedback from professors helps us see that we made a mistake. So, if we work in the real situation and a nurse warns us, we’ll be more accepting. We understand how to cope with it and how to improve the situation the next time” [C4-D].

Focus group discussions also revealed that experiential learning through diverse modes of learning, particularly through the use of 3D computer–based or VR-based SimBIE scenarios and debriefing, contributed to participant learning as evidenced by their progression through Kirkpatrick’s levels 1 to 3. Level 1 (reaction) demonstrated participant satisfaction, engagement, and perceived relevance of the interventions. Level 2 (learning) revealed improvements in participants’ knowledge, skills, attitudes, and confidence. Level 3 (behavior) highlighted the effect of game practice and debriefing on participants’ subsequent behavior. Refer to Multimedia Appendix 7 for the themes derived from the qualitative analysis of focus group discussions, conducted using Kirkpatrick’s model to evaluate the intervention (movies, MOOCs, and 3D computer–based or VR-based SimBIE) across study arms A, B, and C.

Concerning the level 1 of the New World Kirkpatrick model, participants in arm A expressed satisfaction with the 3D simulations, described as “fun and simulates (real situations)” (A3-MT). In arm B, the MOOC and movie were praised for providing “concise briefings on each topic, ... short enough to keep them engaging ... educational and offered a glimpse into real-life situations” (B4-P). VR simulations in arm C were appreciated for being immersive, “fun and realistic” (C1-N). Engagement with the training varied by modality. In arm B, one pharmacist reported periods of inactivity due to limited task engagement, commenting that “... When it finished quickly, delivered and chose the medicine quickly, there were time left ...” (B2-P). However, a nurse (B4-N) expressed that “... in emergencies there will be unexpected events ... there is a skill that will help the patient survive in that situation ... I also practiced critical thinking,” indicating that the participant valued the simulation’s ability to create unexpected challenges, fostering critical thinking skills. For VR simulations in arm C, one pharmacist noted, “... the format in the game made it really tangible ... The sequence of steps was similar to a real situation ...” (C1-P). Participants stated that training relevance was evident, as a radiological technologist mentioned, “Regarding ISBAR communication, ... I feel like this is a lesson that can really be applied” (B1-RT). The feedback highlighted ISBAR communication’s practical applicability based on the participant’s internship experience. While participants acknowledged the training’s alignment with health care practices, they also suggested improving simulated equipment and scenarios to better reflect real-world conditions in the simulation training.

Based on Kirkpatrick’s level 2, feedback from the participants revealed that training improved teamwork, communication, clinical reasoning skills, and confidence. A nurse noted, “We communicated (with other professions). If there was any problem ... I felt more courageous to ask ...” (A5-N). A doctor mentioned, “I practiced taking the patient’s history ... and thinking about what type of disease to diagnose” (A5-D). Participants’ feedback also demonstrated a positive shift in attitudes toward IPC. A pharmacist (C2-P) mentioned that “... there is the issue of relationships among the team members. It will affect the efficiency of the teamwork ...,” emphasizing that positive team dynamics impact effective teamwork. A doctor recognized the shift from physician-led decision-making to a more collaborative approach: “At first, I thought the doctor made every decision ... but the movie changed that mindset” (C1-D). Training also built confidence in patient safety protocols. A radiological technologist remarked, “I was brave to point it out ... to recheck” (B3-RT). Repeated practice helped reinforce learning, as a doctor from arm B reflected

... It was a progress where I already knew mistakes from the first time, and I wouldn’t forget for the second time. I feel that, in real life, if I have ever encountered something similar ... that I knew a little bit before actually seeing the patient, it would be better and make me more confident.

B5-D

This comment showed a sense of progress and preparedness to tackle challenges encountered during the patient treatment.

Participant reflections also revealed that iterative practice and debriefing drove behavioral improvements, in relation to the level 3 of the New World Kirkpatrick model. A nurse shared, “As I kept playing over and over, I learned from mistakes and did better” (C1-N). A radiological technologist stressed that practical experience was crucial for translating theoretical knowledge into action: “... When it comes to playing VR, ... I feel that experimenting, debriefing, or reflecting after playing for the first time is quite effective ... When I came to play round 2, I felt that errors hardly occurred at all” (B1-RT). Debriefing played a pivotal role in self-assessment and team learning. A doctor reflected, “I knew it was wrong, but I wanted to know how to fix it and make it better” (B5-D). A medical technologist noted, “Debriefing prepares us to enter good teamwork in the future” (B4-MT).

Beyond individual reflection, debriefing strengthened team cohesion. A radiological technology student highlighted, “It’s not just about making mistakes visible ... we brainstormed together to solve problems” (C5-RT). These insights underscore debriefing as essential for learning, collaboration, and real-world readiness.

## Discussion

### Principal Findings

This study demonstrated that an innovative IPE curriculum—integrating medical movies, MOOCs, and the ER-VIPE platform using 3D computer–based or VR-based SimBIE with co-debriefing—significantly enhanced TeamSTEPPS performance across all 5 domains. These improvements were reflected in higher mTPOT scores among undergraduate clinical students from 5 disciplines compared to those in the control group, who received 3D computer–based SimBIE without prior preparation or debriefing. Qualitative findings, analyzed through the TeamSTEPPS framework, supported these results. Focus group discussions, interpreted using the New World Kirkpatrick model (levels 1-3), revealed positive impacts on learner satisfaction, engagement, knowledge, skills, attitudes, and confidence—reinforced by game-based practice and structured debriefing. Additionally, simulator sickness significantly increased in the VR group (arm C: Δ=21.0; *P*=.001) but not in the computer-based group (arm B: Δ=5.3; *P*=.44).

This IPE simulation curriculum introduced a novel learning approach that surpassed traditional lectures, breaking down professional silos and promoting collaboration across professions. Grounded in Kolb’s experiential learning theory [25], it emphasized learning from mistakes and enhancing team performance, with the potential to reduce medical errors and improve patient safety, particularly in time-sensitive ED scenarios.

### Rater Reliability and Confounders in Team Performance Assessment

This study demonstrated good to excellent interrater reliability agreement, confirming the validity of mTPOT for assessing team performance across professions during interprofessional collaborative simulation training. No significant confounding factors were identified, except for a minor difference in debriefing duration (9 minutes) and increased simulator sickness in the VR group. The study’s double-blind design (involving raters and a statistician), quasi-experimental mixed methods approach, and the adaptation of mTPOT within the TeamSTEPPS framework, tailored to specific scenarios, ensured reliable assessments. The involvement of raters from 5 professions, combined with comprehensive rater training, further strengthened the study’s validity and contributed to the observed improvements in participant outcomes.

### Comparison With Prior Work

#### Impact of 3D Computer–Based or VR-Based SimBIE on TeamSTEPPS Performance

Previous reviews of IPE programs incorporating TeamSTEPPS and collaborative emergency care simulations have described varying program durations, ranging from short 3- to 9-hour sessions to full-day events and semester-long formats. These interventions often used standardized patients or high-fidelity mannequins, which are associated with a cost-utility ratio approximately 3-4 times higher than that of computer-based simulated patients [43]. The majority of TeamSTEPPS-based curricula emphasized core communication strategies, including ISBAR for handovers, briefing, Concerned, Uncomfortable, Safety statements to escalate safety concerns, closed-loop communication, and check-backs. Less frequently incorporated elements included situational monitoring tools such as the Status, Team, Environment, Progress model, shared mental models, and feedback acknowledgment [18].

These studies also featured a range of participants, including students and health care professionals. Several focused primarily on medical and nursing students, such as Robertson et al [44], Hobgood et al [45], and Liaw et al [46], and reported outcomes at Kirkpatrick levels 2 and 3—demonstrating improvements in knowledge, attitudes, self-confidence, and perceptions of interprofessional communication and teamwork skills performance. Other studies, such as Brock et al [47], included broader cohorts, engaging students from medicine, nursing, pharmacy, and physician assistant programs, while Clark et al [48] included participants from pharmacy, nursing, social work, and respiratory therapy disciplines [18].

In contrast, our study simultaneously involved students from 5 health professions—clinical medicine, nursing, pharmacy, medical technology, and radiologic technology—within a 5-hour multimodal IPE framework. This included preparatory content via medical movies and MOOCs, a 3D computer–based or VR-based SimBIE, and structured co-debriefing. The inclusion of multiple disciplines more closely replicates the complexity of real-world interprofessional teamwork during clinical emergencies, fostering a better understanding of roles, collaborative decision-making, and effective communication.

Our results demonstrated significant gains across all 5 TeamSTEPPS domains—team structure, communication, leadership, situation monitoring, and mutual support—meeting Kirkpatrick level 3 criteria in terms of changes in teamwork performance. This extends previous research by demonstrating that 3D computer–based or VR-based SimBIE enhances not only interprofessional communication, such as ISBAR and closed-loop communication [49], but also all 5 TeamSTEPPS domains. Prior studies have shown that VR SimBIE can produce communication outcomes comparable to those achieved through high-fidelity physical simulations [50]. These findings support the potential of our approach as a scalable, time- and cost-efficient model for IPE that accommodates large cohorts while requiring minimal logistical and infrastructural resources [51]. Nonetheless, further research is warranted to assess long-term impacts (Kirkpatrick level 4), including sustained improvements in safety culture and patient care outcomes.

This approach to training future health care students may contribute to improved patient outcomes, as supported by prior studies in the literature. Previous research has linked improved TeamSTEPPS performance to better patient safety outcomes, including reductions in medical errors, adverse events, and mortality rates [51-53]. Mohsen et al [51] reported significant decreases in adverse events across diagnosis, treatment, preventive services, and communication errors, along with increased patient satisfaction levels. Borckardt et al [52] presented preliminary data, indicating that hospital TeamSTEPPS implementation, as measured by TPOT, was associated with reduced length of stay and lower mortality index after training. Similarly, Shi et al [53] observed reductions in perioperative death rates, antibiotic use, and critical transfusion incidents.

#### Qualitative Insights on TeamSTEPPS Domains

The simulation reinforced teamwork by helping participants understand team structure, team roles, communication, and collaboration in patient care. Clear communication, especially in emergencies, was crucial, and the simulation enhanced 2-way communication across professions. Participants, particularly those with little leadership experience, gained confidence in leading a team. The SimBIE training fostered teamwork aligning with research on simulation [16] and extended reality’s role in improving IPC [54].

Participants emphasized situational monitoring, proactive engagement, and team coordination in patient care. They recognized the need to assess team availability before communication and act cautiously during procedures. Mutual support fostered a blame-free environment and constructive feedback to improve performance. Overall, the simulation enhanced team clinical reasoning and decision-making in a supportive learning environment. The findings align with previous research, demonstrating the effectiveness of simulation in enhancing clinical decision-making, clinical reasoning, and critical thinking [55,56].

#### Qualitative Analysis Through Kirkpatrick’s Lens

Participants expressed satisfaction with the engaging and experiential nature of the simulation and noted the practical applicability of learned knowledge and skills, particularly communication skills and clinical reasoning. However, some participants faced challenges including difficulty with simulated objects and limited interprofessional interaction in the simulation. These issues are common in interprofessional simulation platforms [57]. Concerning their attitude toward interprofessional health care, they demonstrated the shift from physician-led decision-making to a more collaborative approach. The training notably increased their confidence in effective communication and verification processes, crucial for ensuring patient safety. This aligns with the findings of studies using Kirkpatrick’s evaluation model, which demonstrate the positive impact of interprofessional simulation activities on participant reaction and learning [58,59].

Iterative practice and debriefing sessions were identified as critical components of the learning process, fostering self-assessment, team cohesion, and overall performance enhancement. Participants emphasized the value of these sessions in providing opportunities for reflection on errors, skill improvement through repeated practice and feedback, and collaborative problem-solving. Debriefing sessions facilitated self-reflection, collective learning, and the development of effective strategies for real-world scenarios. These findings align with previous research [58] in highlighting the significance of incorporating iterative practice and debriefing into interprofessional simulation training to optimize learning, promote teamwork, and encourage positive behavioral change.

Beyond previous studies of interprofessional simulations using TeamSTEPPS that primarily measured knowledge and attitudes [60,61], our study evaluated actual team performance. The study by Brock et al [62] showed that an interprofessional simulation-based TeamSTEPPS training involving medical, nursing, pharmacy, and physician-assistant students improved their attitudes and knowledge, reflecting Kirkpatrick’s level 2 in team communication for patient safety. In comparison**,** this study’s results align with Kirkpatrick’s levels 1-3, demonstrating improvements in participant satisfaction, learning, and performance. These findings reinforce the scalability and effectiveness of 3D computer–based or VR-based SimBIE.

#### Critical Role of Movies, MOOCs as Preparatory Learning, and Co-Debriefing in SimBIE

The structured instructional design—incorporating a flipped classroom approach with medical movies, MOOCs for preparation, experiential learning via 3D computer–based or VR-based SimBIE, and co-debriefing in a safe environment—contributed to significant learning gains. Participants who engaged with this innovative IPE model showed notable improvements across all TeamSTEPPS domains.

The mTPOT scores further highlighted the effectiveness of educational media in enhancing team performance before SimBIE practice. Baseline mTPOT scores were significantly lower in arm A than in arms B and C, likely due to the absence of movies and MOOCs before simulation day. The considerable difference in baseline scores suggests that these preparatory tools were powerful in improving TeamSTEPPS performance. By fostering a shared mental model, movies and MOOCs ensured that learners from diverse backgrounds developed a common understanding of teamwork principles and language, enhancing their ability to apply TeamSTEPPS strategies effectively during simulations [63]. Movies and MOOCs play a significant role in preparing prior knowledge, which influences learning outcomes, particularly in experiential learning and the flipped classroom approach. Providing foundational knowledge before simulation-based training is essential for maximizing its effectiveness and enhancing training outcomes [19,20].

Notably, qualitative data suggested that debriefing was the critical component in reinforcing learning and promoting further progress [64]. Participants who received facilitator-led debriefing demonstrated improved scores, while the control group showed no change [65]. This finding aligns with Kolb’s experiential learning theory, which emphasizes learning through reflection on experience and individual mistakes [66]. Participants also reported gaining substantial benefits from the hands-on experiential learning provided by the 3D computer–based or VR-based SimBIE, which complemented their self-study through movies and MOOCs.

#### Facilitator-Led Co-Debriefing: Crucial Role and Implementation Challenges

Facilitator-led co-debriefing played a crucial role in reinforcing TeamSTEPPS principles. While arm A (without debriefing) showed no improvement despite scenario repetition, arms B and C (with debriefing) demonstrated significant gains. This underscores debriefing’s crucial role in reinforcing communication, teamwork skills beyond scenario familiarity, and deliberate practice. These findings align with previous research, highlighting debriefing as a key factor in improving SimBIE learning outcomes, even surpassing the benefits of video-based IPE alone [67].

However, implementing debriefing in IPE requires substantial faculty resources, typically involving 2-5 experienced instructors, which may not be feasible in all settings. While the inclusion of instructors from different professions enhances effectiveness [68], institutional limitations can hinder large-scale implementation. Alternative approaches, such as peer-led debriefing or technology-supported remote co-debriefing, could provide a feasible and sustainable solution to maintain learning outcomes while reducing faculty burden [69,70].

#### Practical Implications and Optimization of VR Versus Computer-Based SimBIE

Compared to physical simulation, 3D computer–based simulation reduces constraints related to space, staffing, and specialized equipment, making it a cost-effective solution for IPE. 3D computer–based or VR-based simulation, now widely adopted across continents, offers standardized, scalable, and cost-effective training and enhances opportunities for IPC through simulated patient interactions [17,71].

A potential ceiling effect in arm C cannot be entirely ruled out. The average pretraining mTPOT score in arm C was 2.88 (95% CI 2.74-3.01), and 2.75 (95% CI 2.62-2.89) in arm B—both moderately high relative to the maximum score of 5. Although these scores were not at the theoretical upper limit, they may have left less room for measurable improvement, especially within a short intervention time frame for undergraduate clinical students. Additionally, participants with stronger baseline performance might have reached a performance plateau. While arm C showed a smaller gain than arm B, the difference was not statistically significant. This should not be interpreted as lower effectiveness of the intervention in arm C, but rather may reflect a ceiling effect that constrained further measurable gains. This limitation should be taken into account when interpreting the magnitude of observed improvements.

This study found no significant differences in TeamSTEPPS competency improvements between VR and computer-based SimBIE. Both modalities resulted in similar gains, particularly in the team structure and communication domains, followed by leadership, situation monitoring, and mutual support. This suggests that both modalities can be effective for training teamwork and communication skills. While VR offers an immersive experience and a realistic and engaging learning environment [72], computer-based SimBIE is more practical due to its ease of use, lower incidence of visually induced motion sickness [73], reduced technological demands and cost, and greater scalability. These advantages make computer-based SimBIE a more feasible option for large-scale implementation.

### Limitations

This study was conducted at a single academic institution using a locally developed simulation platform (SimBIE) and involved a relatively small, homogeneous group of clinical Thai students. These factors may limit the external validity of the findings and their generalizability to other educational institutions, international clinical settings, or resource-constrained environments.

This study used a stratified convenience sampling approach instead of randomization due to scheduling constraints during the pandemic, which may have introduced potential selection bias. Group differences could have potentially confounded the outcomes, despite statistical control. Statistical comparisons using the Fisher exact test and the Kruskal-Wallis *H* test showed no significant pre-existing differences in age, gender, academic year, or performance between the study arms. Additionally, none of the participants had received prior TeamSTEPPS training, as such content is not included in undergraduate health care curricula in Thailand. This helped reduce the likelihood of confounding due to prior knowledge or motivation.

Although efforts were made to implement a double-blinding process, the distinct formats of the interventions (VR vs 3D computer SimBIE) may have led participants to infer their group assignment. This may have introduced performance or expectancy bias, particularly in subjective self-assessment and perceived team interaction outcomes, as some participants may have experienced reduced confidence or morale regarding their team’s performance upon suspecting their group allocation.

We acknowledge that the posttest was conducted immediately after the simulation and debriefing, meaning that many observed behaviors were likely stored in short-term memory. Designing SimBIE scenarios to accommodate diverse learners posed challenges. While collaborative teamwork improved, some roles, such as clinical pharmacists and medical technologists, had limited engagement. Pharmacists were often excluded from negative pressure rooms, reducing interprofessional communication, while medical technologists had to wait for orders, restricting active participation. Additionally, potential rater unblinding may have occurred due to differences in movement patterns between 3D computer–based and VR-based applications. While raters could identify group C participants, they remained blinded to pre- and postassessments and the distinction between arms A and B.

The qualitative component of this study was constrained by limited time and resources, which affected the depth of data validation and precluded formal triangulation, recognized as a limitation of our research design. While we relied solely on focus group interviews for qualitative data collection, informal member checking was conducted during the sessions to enhance credibility. We acknowledge that the use of a single qualitative method may limit the trustworthiness of our findings. Nonetheless, we sought to ensure internal consistency by comparing themes from the qualitative data, with trends observed in the quantitative analysis, which revealed consistent responses across both data sources.

### Future Direction

This study demonstrates the effectiveness of a novel IPE model using a TeamSTEPPS-based approach that integrates medical movies, MOOCs, and 3D computer–based or VR-based SimBIE with co-debriefing to strengthen collaborative practice among health care students. To build on these findings, future research should assess long-term retention and real-world impact and expand to multicenter trials with diverse learner populations to enhance generalizability across different educational and clinical contexts. Integrating this model across all educational levels and including additional health professions such as paramedics and emergency medical technicians may further strengthen health care team culture, improve patient safety, social return on investment, and enhance system-wide crisis response.

To support broader adoption, we are pursuing several key initiatives aimed at enhancing equity, applicability, and accessibility of the SimBIE platform. We are currently awaiting intellectual property approval and have initiated a spin-off business model to enable equitable global dissemination through affordable pricing, cloud-based delivery, system maintenance, and user support. In terms of language equity, the platform interface, simulation content, and medical edutainment materials are designed to be multilingual-ready, with the initial version available in both Thai and English. This helps reduce language barriers and supports broader regional and international adoption. We recognize that equity, in this context, involves not only platform access but also the feasibility of implementation across diverse educational and resource-constrained environments. To address this, we are developing a Train-the-Trainer model to build capacity in regional academic hubs. This approach enables faculty—particularly those in underserved areas—to receive training in facilitation and co-debriefing, allowing them to independently implement the SimBIE+TeamSTEPPS model in their own institutions with high fidelity.

We recognize that equity extends beyond access to technology and includes the openness of knowledge dissemination. Limiting SimBIE to a paid subscription model may conflict with academic principles of inclusivity and open collaboration. To balance these concerns, we provide key pedagogical frameworks, SimBIE flow designs, and assessment tools (eg, mTPOT and SSQ) within the manuscript to support reproducibility, even for those without direct platform access. This blended model seeks to balance academic value with long-term sustainability through a feasible business approach, ensuring both global scalability and equitable impact.

Our approach is also aligned with the WHO Global Strategy on Digital Health, which emphasizes strengthening health systems through digital innovation to advance the goal of “Health for All” [74]. As national policies increasingly promote IPE and TeamSTEPPS for improving 2P Safety, digital solutions like ER-VIPE offer scalable and impactful support for widespread implementation.

Regarding scalability, we are transitioning to a cloud-based annual subscription model, allowing institutions to access the platform without the need for substantial infrastructure investment. We are also seeking government research funding and collaborating with national policy makers, health professional councils in Thailand (eg, the Medical Council, Nursing Council, Pharmacy Council, and Allied Health Council), and key stakeholders—including the Ministry of Public Health, the HAI of Thailand, the Royal College of Emergency Physicians of Thailand, and the National Institute for Emergency Medicine. Key strategies include nationwide implementation of ER-VIPE in health care settings, official accreditation for continuing professional education, and integration into university-level health curricula. These efforts aim to institutionalize ER-VIPE within national education and patient safety systems, ensuring widespread adoption and policy-level impact. Ultimately, this will promote IPC and patient safety based on TeamSTEPPS and IPE principles while supporting long-term sustainability and scalability—first nationally, then internationally.

To further support sustainability, we are developing artificial intelligence (AI)–assisted tools, including personalized AI for competency training, automated assessments, AI-supported co-debriefing, and electroencephalography-integrated feedback in future versions. These innovations aim to reduce reliance on high-burden human facilitators and enable continuous, scalable implementation, particularly in low-resource settings. Collectively, these efforts strengthen equity, scalability, and long-term sustainability—positioning SimBIE for nationwide rollout and global dissemination, especially in resource-limited settings. This vision is essential to prepare the future health care workforce to thrive in a volatile, uncertain, complex, and ambiguous world.

### Conclusions

This study presents an effective and scalable model for TeamSTEPPS performance training by integrating medical movies, MOOCs, and 3D computer–based or VR-based SimBIE with co-debriefing. Aligned with Kolb’s experiential learning theory, the approach highlights the benefits of experiential and multimedia-based learning preparation in a flipped classroom. Based on the positive results within this specific context, the research team suggests that this model shows promise for enhancing preparedness among clinical undergraduate multiprofessional students for ED practice and for ultimately improving 2P Safety and patient outcomes. Further studies are needed to confirm its generalizability and feasibility across diverse institutions before recommending widespread adoption.

## Appendix

Multimedia Appendix 1 CONSORT checklist.

Multimedia Appendix 2 Team Strategies and Tools to Enhance Performance and Patient Safety (TeamSTEPPS) scoring (Modified TeamSTEPPS Team Performance Observation Tool) for physicians.

Multimedia Appendix 3 COREQ checklist.

Multimedia Appendix 4 Participant demographics by specialty.

Multimedia Appendix 5 Baseline Modified TeamSTEPPS (Team Strategies and Tools to Enhance Performance and Patient Safety) Team Performance Observation Tool scores.

Multimedia Appendix 6 Percentage agreement and weighted κ statistics by profession during pre and postfinal assessments.

Multimedia Appendix 7 Themes from qualitative analysis of focus group discussions using Kirkpatrick’s model on the intervention (movies, massive open online courses, and virtual simulation) across study arms A, B, and C.

## Abbreviations

2P Safety: personal and patient safety
AI: artificial intelligence
CONSORT: Consolidated Standards of Reporting Trials
COREQ: Consolidated Criteria for Reporting Qualitative Research
ED: emergency department
ER-VIPE: Emergency Room-Virtual Interprofessional Education
HAI: Healthcare Accreditation Institute
IPC: interprofessional collaboration
IPE: interprofessional education
ISBAR: Identify, Situation, Background, Assessment, and Recommendation
MOOC: massive open online course
mTPOT: Modified TeamSTEPPS Team Performance Observation Tool
SimBIE: simulation-based interprofessional education
SSQ: Simulator Sickness Questionnaire
TeamSTEPPS: Team Strategies and Tools to Enhance Performance and Patient Safety
TPOT: Team Performance Observation Tool
VR: virtual reality
WHO: World Health Organization

## Data Availability

The datasets generated or analyzed during this study are not publicly available due to institutional review board restrictions on participant privacy and data security but are available from the corresponding author on reasonable request.
