# Supplementary material for: Enhancing Team Strategies and Tools to Enhance Performance and Patient Safety Performance Through Medical Movies, Massive Open Online Courses, and 3D Virtual Simulation–Based Interprofessional Education: Mixed Methods Double-Blind Quasi-Experimental Study
Source: J Med Internet Res. 2025 Sep 8;27:e67001. doi: 10.2196/67001 (PMC12455160; doi:10.2196/67001)
Supplement: Multimedia Appendix 2 [file jmir_v27i1e67001_app2.pdf]

Team STEPPS scoring for physician

Email

Rater name (e.g. XXX)

Rater No. (e.g., D01)

Study code (e.g. D01_Gr28)

VDO link that you watched XXX

Scoring Criteria

5= Excellent, perfect performance, consistently appropriate in every situation

4= Good/ mostly perform

3= Should perform more often/ more consistently but is acceptable

2= Poor/ should perform more often

1= Very poor/should perform more often but never perform

1. Team structure

1.1. Team meets up

The observed behavior in SIM

There is a brief with pressing the group call button, whether for making a call or answering an incoming call, from the beginning of playing games.

- ○ (5) There is a brief with pressing a group call button within the first 5 minutes of the game
- ○ (4) There is a brief with pressing a group call button more than 5 minutes after starting the game, but within (5 to <10 minutes.)
- ○ (3) There is a brief with pressing a group call button more than 10 minutes after starting the game, but within (5 to <10 minutes.)
- ○ (2) There is a brief with pressing a group call more than 15 minutes after starting the game or pressing the group call without any brief
- ○ (1) There is no group call

1.2. Determine the role and responsibility of team members

The observed behavior in SIM

There is a pressing group call button to introduce one's name and clear role and responsibilities (Clear role & goal).

The physician introduces themselves by stating their name, profession, and responsibilities.

- ○ (5) The physician introduces themselves by stating their name, position, and responsibilities. (3/3)

- (4) The physician introduces themselves by stating their name, position, and responsibilities, but not clear or accurate. (3/3)
- (3) The physician introduces themselves by stating at least 2 out of 3: name, position, and responsibilities. (2/3)
- (2) The physician introduces themselves by stating at least 2 out of 3: name, position, and responsibilities, but it may not be clear or accurate. Or they introduce themselves by stating only 1 out of 3: name, position, and responsibilities. (2/3 or 1/3)
- (1) The physician does not introduce themselves by stating their name, position, and responsibilities. (0/3)

1.3. Delegating responsibility to team members

The observed behavior in SIM

1)There are tasks assigned to the airway nurse to prepare airway equipment and monitor O2 saturation during or after intubation.

2)There are tasks assigned to the circulating nurse to help with venipuncture, open lines, and medication.

3)There is discussion or consideration of other individuals who are not part of our team, such as calling for help, or consulting various specialists, for example, contacting an anesthesiologist, ENT specialist, ICU, or infectious disease specialist.

- (5) The physician can perform all three tasks clearly and within an appropriate time. (3/3)
- (4) The physician can perform all three tasks, but not clearly or within an appropriate time. (3/3)
- (3) The physician can perform 2 out of 3 tasks clearly and within an appropriate time. (2/3)
- (2) The physician can perform 1 out of 3 tasks or 2 out of 3 tasks, but it may not be clear and not within an appropriate time. (1/3 or 2/3)
- (1) The physician does not assign tasks or initiate a call for help. (0/3)

1.4. Involving patients and their families as part of the team.

The observed behavior in SIM

1)Allowing patient involvement in decision-making and consent for intubation or tracheostomy before the medical procedure (inform & verbal consent)

2)Assigning team members to do patient history taking, including drug allergies, medication use, and previous hospitalizations, from the relatives.

- (5) There ask for informed consent from the patient, and team members do a history taking about drug allergies and hospitalization history directly from the patient or their family. There also asked for the patient and family's opinions on any special requests or concerns, such as if the intubation is too difficult or do not want to proceed. (2/2)
- (4) There ask for informed consent from the patient, or team members to do a history taking about drug allergies and hospitalization history directly from the patient or their family. However, it is not an appropriate time, or the information is incomplete. (2/2, but 1/2 done poorly)

- (3) There ask for informed consent from the patient, and team members do a history taking about drug allergies and hospitalization history directly from the patient or their family. However, it is not an appropriate time, or the information is incomplete. (2/2, but 2/2 done poorly)
- (2) Lack of either asking for informed consent from the patient or doing history taking from the family by the team (1/2)
- (1) Lack of both asking for informed consent from the patient or doing history taking from the family by the team (0/2)

2. Communication

2.1. Communicating information among team members, concisely, and correctly at an appropriate time

The observed behavior in SIM

Communicate with Clear Concise Correct is communication information.

Clear

Concise

Correct ex. informs the critical numerical value, units, and testing procedure.

(5) The physician can always perform all three tasks clearly and correctly or more than 80% of the time. (3/3)

(4) The physician can perform all three tasks in more than 50% of the instances, but either at an inappropriate time or with a reluctant tone of voice. (3/3)

(3) The physician can perform two out of three tasks clearly and correctly every time. (2/3)

(2) The physician can perform one out of three tasks or two out of three tasks, but it is still unclear or incomplete. (1/3 or 2/3)

(1) The physician does not perform all three tasks. (0/3)

2.1. Searching information from existing sources

The observed behavior in SIM

1)From the patient

2)The expert team pressed the call for help button.

3)Looked up the Evidence-Based Medicine (EBM) guideline.

4)Consulted the Medical Technologist (MT) about Lab i.e. collection/exporting, and Specimen result interpretation

5)Consulted the pharmacist about medication

6)Accessed Electronic Medical Records (EMR) for information such as medical history, medication allergies, and COVID-19 risk.

- (5) Consulted with all existing information sources or 5-6 sources (5/6-6)
- (4) Consulted with just 4 existing information sources (4/6)
- (3) Consulted with just 3 existing information sources (3/6)
- (2) Consulted with 1-2 existing information sources (1-2/6) or searched unsuccessfully such as being unable to receive accurate information
- (1) Did not search from any information sources (0/6)

2.2. Utilize check-back technique to verify information among team communication

The observed behaviors in SIM

There is always of check-back technique in communication, especially for:

1) Patient's name
2) Medication name
3) Medication dose
4) Medication route
5) Medication rate
6) Critical value K (with specified units)

- (5) There is always a check-back with a complete 6 information when necessary(6/6)
- (4) There is a check-back for 4-5 out of 6 information (4-5/6)
- (3) There is a check-back for 3 out of 6 information (3/6)
- (2) There are barely any check-backs or lack of check-backs for essential information (1-2/6)
- (1) There are no check-backs during communication (0/6)

2.4. Utilize the ISBARR technique, call-outs, and handover for effective communication among team members.

The Observed Behaviors in SIM:

1) The team consistently uses ISBARR to communicate each time:
    1. Introduction: Introduce themselves and provide patient identification, including at least 2 out of 3 pieces of information (name, hospital number, date of birth).
    2. Situation
    3. Background
    4. Assessment
    5. Recommendation/Request
2) In emergencies, the team leader call-outs for Airway, Breathing, and Circulation, communicating with the nursing team to confirm the completion of each step.

- (5) There is a performance of both actions properly.(2/2)
- (4) There is a performance of both actions but not properly.(2/2)
- (3) There is a performance of only one out of two actions properly
- (2) There is a performance of only one out of two actions but not properly.
- (1) Neither actions are performed, or there are severe consequences due to miscommunication during handover (0/2).

3. Leadership

3.1. Set the team's goals and vision

The observed behavior in SIM

There is a pressing the group call button to brief or huddle and report the issues and goals to the team clearly from the beginning.

- (5) There is a pressing the group call button to brief or huddle and report the issues and goals to the team clearly from the beginning (within 5 minutes)
- (4) There is a pressing the group call button to brief or huddle and report the issues and goals to the team clearly after starting the game within 10 minutes
- (3) There is a pressing the group call button to brief or huddle and report the issues and goals to the team clearly after 10 minutes starting of the game
- (2) There is a pressing the group call button to brief or huddle and report the issues and goals to the team clearly after 15 minutes starting of the game, or there is a group call but the information and issues are unclear or inaccurate
- (1) There is no pressing the group call to brief or huddle and report the issues and goals to the team.

3.2. Utilize resources efficiently to maximize team productivity

The observed behavior in SIM

1) Able to make decisions to assist the patient with LMA insertion or perform neck puncture until reaching a normal O2 saturation within 4 minutes of entering the negative pressure room (effectively prioritizing time management).
2) Consult an ENT specialist or a medical expert in medicine inhalation after being informed about difficult airway history or at least two failed efforts at intubation. (Well-managed team members' contingency by an early call for help)
3) Inform the airway nurse to be prepared with airway aid equipment by ensuring about tube's number, the lubrication gel, and batteries.

- (5) The physician can effectively and accurately perform all three tasks within an appropriate time (3/3).
- (4) The physician can perform all three tasks (3/3), without completion, such as informing the nurse with insufficient detail, etc.
- (3) The physician can perform two out of three tasks completely, accurately, and within an appropriate time (2/3).
- (2) The physician can perform one out of three tasks or two out of three tasks, but not completely and accurately.
- (1) The physician does not perform all three tasks (0/3).

3.3. Preserve balance and distribute responsibility in a team equally and appropriately

The observed behavior in SIM

1) While intubating, there is a cooperation between the physician and nurse in the:
   - Asking for consent
   - Preparing equipment
   - Positioning
   - Monitoring the patient's vital signs
   - Squeezing the Ambu bag
2) The physician observes the team's work, provides feedback, redistributes or assigns tasks, and encourages team members appropriately. (***Balance means everyone is active, and in reality, that person can help other colleagues with various tasks)

- (5) There is a balance and cooperation between the physician and nurse in managing the airway in all aspects, and there is an observation of assigned tasks among team members with appropriate feedback and encouragement. (2/2)
- (4) Both tasks are performed, but there is a mistake in one of the tasks. (2/2, but 1/2 not done well)
- (3) Both tasks are performed, but there are mistakes in both tasks. (2/2, but 2/2 not done well)
- (2) One of the tasks is not performed. (1/2)
- (1) Neither of the tasks is performed. (0/2)

3.4. Assigning tasks and responsibilities appropriately

The observed behavior in SIM

1) Able to distribute tasks to airway nurse, and nurse circulation appropriately (physician shouldn't provide medication to the patients especially when there is no independent double check)
2) The physician performs ABG puncture and sends it or assigns the nurse to deliver the ABG specimen accurately (ABG is a physician duty, such as puncturing and discarding the needle, attaching stickers, and checking the patient's wristband before attaching, these should assign to the nurse).
3) Able to assign consultation to the nurse to prepare the patient's transport equipment with the negative chamber accurately.

(5) The physician can perform all three tasks, accurately, and within an appropriate time (3/3).

(4) The physician can perform all three tasks (3/3), but they are not clear, accurate, or within an appropriate time.

(3) The physician can perform two out of three tasks, accurately, and within an appropriate time (2/3).

(2) The physician can perform only one out of three tasks, or they can perform two out of three tasks but not, accurately, or within an appropriate time.

(1) The physician does not assign any tasks and does not deliver tasks they are responsible for (0/3).

3.5. There is a practice of conducting briefings, huddles, and debriefings. The observed behavior in SIM

1) There is a group call before entering the negative pressure room (briefing).
2) There is a group call inside the negative pressure room (huddle).
3) There is a group call after leaving the negative pressure room for debriefing.

- (5) There is a group call for briefing, huddling, and debriefing, and clear and helpful communication (3/3).
- (4) There is a group call for briefing, huddling, and debriefing, but there are some communication shortcomings (3/3).
- (3) There is a lack of group calls for briefing, huddling, and debriefing in one aspect (2/3).
- (2) There is a lack of group calls for briefing, huddling, and debriefing in both aspects (1/3).
- (1) There is no group call for briefing, huddling, and debriefing (0/3).\

3.6. Be an example of good teamwork in a medical context.

The observed behavior in SIM

1) Showing respect to team members through polite tone and language, and expressing gratitude (Promotes a supportive and collaborative work environment).
2) Embracing and actively listening to constructive from team members.
3) Being able to manage and resolve conflicts within the team (if any).

- (5) Physician can perform all three behaviors (3/3), or although there are no conflicts, still shows respect to colleagues in behavior 1) and always embrace feedback or suggestions from the team. Receive a full score.
- (4) Physician can perform all three behaviors (3/3), but there are parts for improvement, such as tone of voice or use of language (3/3).
- (3) Physician can perform only 2 out of 3 behaviors.
- (2) Physician can perform only 1 out of 3 behaviors or 2 out of 3 behaviors but still need an improvement such as tone of voice or use of language.
- (1) Physician does not perform all three behaviors (0/3), or their performance is poor and requires significant improvement.

4. Situation Monitoring

4.1. Patient Monitoring

The observed behavior in SIM

The physician actively monitors the patient's vital signs and electrocardiogram (ECG) waveform or assigns this task to the nurse at specific intervals:

1) Before intubation.
2) During intubation.
3) After intubation.
4) Before performing a portable X-ray.
5) After the X-ray.
6) Before transferring the patient out of the negative pressure room.

- (5) The physician consistently monitors the patient's vital signs and electrocardiogram (ECG) waveform at least 5 times as specified (5-6/6) or assigns the task to the nurse.
- (4) (If not assigned to the nurse) The physician partially monitors the patient's vital signs and ECG waveform but not as frequently as desired (4/6 times).
- (3) (If not assigned to the nurse) The physician monitors the patient's vital signs and ECG waveform, but with less frequency (3/6 times).
- (2) (If not assigned to the nurse) The physician monitors the patient's vital signs and ECG waveform only 1-2 times (1-2/6 times).
- (If not assigned to the nurse) The physician does not monitor the patient's vital signs and ECG waveform at all (0/6 times).

4.2. Checking up with team members for safety and mistake prevention * The observed behaviors in the SIM:

1) Assisting each other in monitoring and adjusting PPE attire, especially if the back of the gown is open.

2) Observing stress levels and press to encourage each teammate up to three times. At the end of the game, there will not be anybody depleted of energy.
3) Detecting or observing other team members are about to make unsafe actions, such as not lifting the side rail of the bed, proceeding with an X-ray without checking the wristband, or administering medication without checking the patient's identification.

- ○ (5) The physician can perform all three behaviors appropriately, thoroughly, and completely. (3/3).
- ○ (4) The physician can perform all three behaviors but with some deficiencies, such as someone still having their back gown open
- ○ (3) The physician can perform only 2 out of 3 behaviors in an appropriate, thorough, and complete manner.
- ○ (2) The physician can perform only 1 out of 3 behaviors or perform 2 out of 3 behaviors but not complete.
- ○ (1) The physician does not perform all three behaviors (0/3).

4.3. Checking up on the environment for safety and assessing resources preparation

The observed behaviors in the SIM:

The team can manage the negative pressure room efficiently, including the admission process. They appropriately operate medical equipment, devices, and time management. As a result, the team can assist and transfer critically ill patients from the emergency room to the ICU at a perfect time

- ○ (5) The team can transfer patients from the emergency room to the ICU within 30 minutes.
- ○ (4) The team can transfer patients from the emergency room to the ICU within 40 minutes.
- ○ (3) The team can transfer patients from the emergency room to the ICU within 50 minutes.
- ○ (2) The team can transfer patients from the emergency room to the ICU within 60 minutes while ensuring patient safety.
- ○ (1) The team cannot transfer patients to the ICU within 60 minutes, which either exceeds the time limit or the patient is not safe, such as a dead outcome from anaphylaxis (game over).

4.4. Evaluating supervision outcomes and identifying potential changes that may require adjustments in the treatment plan.

The observed behaviors in SIM:

There is a follow-up on the team members' performance to assess the operation of the planned goals and objectives that ensure patient and team safety through face-to-face discussions, personal communication, or group calls while monitoring:

1) The airway nurse's work (vital signs, ventilator tubing).
2) The circulation nurse's work (IV administration, antibiotics, calcium gluconate, lab sample submission).
3) The Radio Tech's work (exposure, resolution of x-rays, result interpretation).
4) The Med Tech's work (lab results, prioritization of different lab tests).
5) The pharmacist's work (verifying medication type, dose, route, rate, medication, checking medication allergies, medical reconciliation).

- ○ (5) There is a follow-up on the work of all team members, covering all 5 parts (5/5).

- (4) There is a follow-up on the work of team members, covering 4 out of 5 parts (4/5).
- (3) There is a follow-up on the work of team members, covering 3 out of 5 parts (3/5).
- (2) There is a follow-up on the work of team members, covering 1-2 out of 5 parts (1-2/5).
- (1) There is no follow-up on the work of team members (0/5).

4.5. There is effective communication to ensure that everyone in the team has a mutual understanding of the problem situations and solutions.

The observed behavior in SIM:

There is sharing of situational awareness (SA) through communication (Think aloud) among relevant team members, enabling them to build a mutual understanding of the problems and various solutions. For example

1) COVID pneumonia risk (Hx travel, positive rapid test result)
2) Difficult airway (HX, PE), Can't ventilate, can't intubate (CVCI)
3) Hyper K 7 mmol/dL
4) Drug allergies
5) Risk of healthcare-associated pneumonia (HAP) due to ICU stay
6) Sharing about differential diagnoses such as COPD with acute exacerbation, heart failure, pneumothorax, etc.
7) Risk of falls from bed
8) Risk of dislodged tubes
9) Identification errors during X-rays, medication administration, blood gas sampling, etc.

- (5) There is sharing of situational awareness (SA) that build a mutual understanding of the problems and various solutions appropriately covering 8-9 points (8-9/9).
- (4) There is sharing of situational awareness (SA) of 6-7 points (6-7/9).
- (3) There is sharing of situational awareness (SA) of 4-5 points (4-5/9).
- (2) There is sharing of situational awareness (SA) of 1-3 points (2-3/9), or there is an improvement in communication when sharing situational awareness.
- (1) There is no or insufficient sharing of situational awareness (SA), with 0-1 point (0-1/9), or there are severe communication mistakes leading to negative outcomes.

5. Support and Assistance

5.1. There is mutual support and assistance in tasks.

The observed behavior in SIM:

1) Helping each other in raising the patient's bed.
2) Helping each other in donning personal protective equipment (PPE).
3) Verbalizing voluntary assistance and support among team members.

- (5) The physician performs all 3 actions appropriately (3/3).
- (4) The physician performs all 3 actions, but not completely (3/3).
- (3) The physician performs only 2 out of 3 actions.

- (2) The physician performs only 1 out of 3 actions or 2 out of 3 actions incompletely.
- (1) The physician does not perform all 3 actions (0/3).

5.2. Giving constructive feedback and appropriate suggestion to team members

The observed behaviors in SIM:

Giving constructive feedback at appropriate times, such as saying expressing gratitude when

1) Team members assist in raising the patient's bed.
2) Assisting in adjusting PPE.
3) Assisting in repositioning the patient during an X-ray or doing an X-ray for the patient.
4) Assisting in notifying critical lab results.
5) Providing consultation regarding medication/allergies, and so on

- (5) Provides constructive feedback and express appropriate gratitude whenever team members assist, with both quantity and quality being complete and accurate.
- (4) Provides constructive feedback and express appropriate gratitude when team members assist, but there are some improvements or performances that are still not as adequate as they should be (performed adequately in terms of quantity and quality).
- (3) Provides feedback and express gratitude when team members assist, but it is less or there still needs an improvement, such as feedback that is not constructive or not provided at the right time (should be done more frequently/consistently).
- (2) Provides feedback and express gratitude when team members assist, but it is less or there still needs an improvement, such as feedback that is not constructive or not provided at the right time (requires significant improvement in both quantity and quality).
- (1) Does not provide constructive feedback or express gratitude to team members at all.

5.3. Uses non-confrontational reminder concepts and the CUS framework to promote patient safety.

The observed behaviors in the SIM include:

1) Reminding to address low oxygen levels (respiratory failure)
2) Reminding to monitor EKG signs of hyperkalemia
3) Reminding to be cautious of dislodged breathing tubes during X-ray or patient movement
4) Reminding us to be careful of COVID-19
5) Reminding to be aware of HAP
6) Reminding to prevent falls from the bed
7) Reminding to avoid administering incorrect or allergenic medications
8) Reminding to prevent identification errors during X-ray, medication administration, or blood gas sampling
9) Reminding to be cautious of difficult airway situations, prepare smaller-sized LMA/ETT equipment or neck puncture devices, and reposition the patient's position
10) Reminding about communication without Call back, or failing to follow the ISBAR.

- (5) Effectively use non-confrontational reminder techniques and CUS framework, with at least 9 out of 10 reminders given (9-10/10).

- (4) Use non-confrontational reminders and the CUS framework, with 7-8 out of 10 reminders given (7-8/10).
- (3) Shows some effort in using non-confrontational reminders and the CUS framework, with 5-6 out of 10 reminders given (5-6/10).
- (2) Shows less use of non-confrontational reminders and the CUS framework, with 3-4 out of 10 reminders given (3-4/10).
- (1) Does not use or even show non-confrontational reminders and the CUS framework, with 0-2 out of 10 reminders (0-2/10).

5.4. Using the two-challenge rule or the DESC technique to resolve conflicts.

The observed behaviors in the SIM include:

1) Issuing a second verbal warning if team members do not hear or fail to take action.
2) Applying the DESC technique to resolve conflicts, if present.

- (5) There is always applying of the two-challenge rule if team members do not hear or take action, and the DESC technique is employed appropriately and effectively (2/2).
- (4) There is always applying of the two-challenge rule if team members do not hear or take action, and the DESC technique is employed when conflicts arise (2/2), but there are some improvements in its proper application.
- (3) There is always applying of the two-challenge rule if team members do not hear or take action, and the DESC technique is employed when conflicts arise (1/2), and its application is activated correctly and effectively.
- (2) There is always applying of the two-challenge rule if team members do not hear or take action, and the DESC technique is employed when conflicts arise (1/2), but there are some improvements in its proper application.
- (1) There is always applying of the two-challenge rule if team members do not hear or take action, and the DESC technique is employed.
