# Supplementary material for: Enhancing Team Strategies and Tools to Enhance Performance and Patient Safety Performance Through Medical Movies, Massive Open Online Courses, and 3D Virtual Simulation–Based Interprofessional Education: Mixed Methods Double-Blind Quasi-Experimental Study
Source: J Med Internet Res. 2025 Sep 8;27:e67001. doi: 10.2196/67001 (PMC12455160; doi:10.2196/67001)
Supplement: Multimedia Appendix 3 [file jmir_v27i1e67001_app3.docx]

| **Topic** | **Item no.** | **Guide questions/description** | **Reported on page no.** |
| --- | --- | --- | --- |
| **Domain 1: research team and reflexivity** | | | |
| *Personal characteristics* | | | |
| Interviewer/facilitator | 1 | Which author/s conducted the interview or focus group? | 11 |
| Credentials | 2 | What were the researcher’s credentials? e.g., PhD, MD | 11 |
| Occupation | 3 | What was their occupation at the time of the study? | 11 |
| Gender | 4 | Was the researcher male or female? | 11 |
| Experience and training | 5 | What experience or training did the researcher have? | 11 |
| *Relationship with participants* | | | |
| Relationship established | 6 | Was a relationship established prior to study commencement? | 11 |
| Participant knowledge of the interviewer | 7 | What did the participants know about the researcher? e.g., personal goals, reasons for doing the research | 11 |
| Interviewer characteristics | 8 | What characteristics were reported about the interviewer/facilitator? e.g., bias, assumptions, reasons and interests in the research topic | 11 |
| **Domain 2: study design** | | | |
| *Theoretical framework* | | | |
| Methodological orientation and theory | 9 | What methodological orientation was stated to underpin the study? e.g., grounded theory, discourse analysis, ethnography, phenomenology, content analysis | 12 |
| *Participant selection* | | | |
| Sampling | 10 | How were participants selected? e.g., purposive, convenience, consecutive, snowball | 11 |

**Multimedia Appendix 3 (continued):** COREQ (COnsolidated criteria for REporting Qualitative research) Checklist

| **Topic** | **Item no.** | **Guide questions/description** | **Reported on page no.** |
| --- | --- | --- | --- |
| **Domain 2: study design** | | | |
| *Participant selection* | | | |
| Method of approach | 11 | How were participants approached? e.g., face-to-face, telephone, mail, email | 11 |
| Sample size | 12 | How many participants were in the study? | 11 |
| Non-participation | 13 | How many people refused to participate or dropped out? Reasons? | N/A |
| *Setting* | | | |
| Setting of data collection | 14 | Where was the data collected? e.g., home, clinic, workplace | 11 |
| Presence of non-participants | 15 | Was anyone else present besides the participants and researchers? | 11 |
| Description of sample | 16 | What are the important characteristics of the sample? e.g., demographic data, date | 11 |
| *Data collection* | | | |
| Interview guide | 17 | Were questions, prompts, guides provided by the authors? Was it pilot tested? | 11 |
| Repeat interviews | 18 | Were repeat interviews carried out? If yes, how many? | N/A |
| Audio/visual recording | 19 | Did the research use audio or visual recording to collect the data? | 12 |
| Field notes | 20 | Were field notes made during and/or after the interview or focus group? | N/A |
| Duration | 21 | What was the duration of the interviews or focus group? | 11 |
| Data saturation | 22 | Was data saturation discussed? | N/A |
| Transcripts returned | 23 | Were transcripts returned to participants for comment and/or correction? | N/A |

**Multimedia Appendix 3 (continued):** COREQ (COnsolidated criteria for REporting Qualitative research) Checklist

| **Topic** | **Item no.** | **Guide questions/description** | | | **Reported on page no.** |
| --- | --- | --- | --- | --- | --- |
| **Domain 3: analysis and findings** | | | | | |
| *Data analysis* | | | | | |
| Number of data coders | 24 | | How many data coders coded the data? | 12 | |
| Description of the coding tree | 25 | | Did authors provide a description of the coding tree? | 12 | |
| Derivation of themes | 26 | | Were themes identified in advance or derived from the data? | 12-13 | |
| Software | 27 | | What software, if applicable, was used to manage the data? | 12 | |
| Participant checking | 28 | | Did participants provide feedback on the findings? | N/A | |
| *Reporting* | | | | | |
| Quotations presented | 29 | | Were participant quotations presented to illustrate the themes/findings? Was each quotation identified? e.g., participant number | 20-23 | |
| Data and findings consistent | 30 | | Was there consistency between the data presented and the findings? | 20-23 | |
| Clarity of major themes | 31 | | Were major themes clearly presented in the findings? | 20-21 | |
| Clarity of minor themes | 32 | | Is there a description of diverse cases or discussion of minor themes? | N/A | |
