# Supplementary material for: Enhancing Team Strategies and Tools to Enhance Performance and Patient Safety Performance Through Medical Movies, Massive Open Online Courses, and 3D Virtual Simulation–Based Interprofessional Education: Mixed Methods Double-Blind Quasi-Experimental Study
Source: J Med Internet Res. 2025 Sep 8;27:e67001. doi: 10.2196/67001 (PMC12455160; doi:10.2196/67001)
Supplement: Multimedia Appendix 4 [file jmir_v27i1e67001_app4.docx]

| **Factors** | **Total (n=87)** | **Medicine (n=15)** | **Nurse**  **(n=30)** | **Pharmacy (n=15)** | **Radiology (n=12)** | **Medical Tech (n=15)** |
| --- | --- | --- | --- | --- | --- | --- |
| **Gender** |  |  |  |  |  |  |
| Female | 60 (69%) | 4 (27%) | 29 (97%) | 7 (47%) | 8 (67%) | 12 (80%) |
| Male | 27 (31%) | 11 (73%) | 1 (3%) | 8 (53%) | 4 (33%) | 3 (20%) |
| **Age** |  |  |  |  |  |  |
| Mean (SD) | 21.87 (1.16) | 22.47 (0.74) | 21.27 (0.83) | 23.47 (1.19) | 21.42 (0.51) | 21.27 (0.46) |
| Median (IQR) | 22 (21-22) | 22 (22-23) | 21 (21-22) | 23 (23-24) | 21 (21-22) | 21 (21-22) |
| **Year** |  |  |  |  |  |  |
| 3 | 13 (15%) | - | 13 (43%) | - | - | - |
| 4 | 46 (53%) | - | 17 (57%) | 2 (13%) | 12 (100%) | 15 (100%) |
| 5 | 18 (21%) | 12 (80%) | - | 6 (40%) | - | - |
| 6 | 10 (11%) | 3 (20%) | - | 7 (47%) | - | - |
| **Academic grade** |  |  |  |  |  |  |
| Mean (SD) | 3.22 (0.38) | 3.34 (0.44) | 3.20 (0.28) | 3.20 (0.46) | 3.25 (0.40) | 3.11 (0.38) |
| Median (IQR) | 3.24 (2.98-3.50) | 3.44 (3.06-3.70) | 3.22 (3.00-3.43) | 3.26 (2.96-3.60) | 3.30 (3.09-3.47) | 3.17 (2.78-3.30) |
| **PHQ9 score < 9** |  |  |  |  |  |  |
| No | 4 (5%) | - | - | - | 2 (17%) | 2 (13%) |
| Yes | 83 (95%) | 15 (100%) | 30 (100%) | 15 (100%) | 10 (83%) | 13 (87%) |
| **PHQ9 score** |  |  |  |  |  |  |
| Mean (SD) | 3.72 (3.04) | 4.20 (2.62) | 1.90 (2.02) | 3.60 (2.59) | 5.42 (2.84) | 5.67 (3.83) |
| Median (IQR) | 4 (1-6) | 4 (2-6) | 1 (0-4) | 4 (1-5) | 5.50 (4-7) | 6 (3-8) |
| **Debrief time (min)** |  |  |  |  |  |  |
| Mean (SD) | 54.96 (12.29) | 56.13 (12.48) | 54.38 (12.37) | 54.11 (13.15) | 57.14 (13.12) | 54.11 (13.19) |
| Median (IQR) | 57 (45-63) | 59 (47.50-66.00) | 56 (45-66) | 56 (45-63) | 60 (46-70) | 57 (45-63) |
| **Debrief staff (person)** |  |  |  |  |  |  |
| Mean (SD) | 5.33 (1.75) | 5.30 (1.83) | 5.30 (1.78) | 5.30 (1.83) | 5.50 (1.85) | 5.30 (1.83) |
| Median (IQR) | 6 (4-6) | 6 (4-6) | 6 (4-6) | 6 (4-6) | 6 (4.50-6.50) | 6 (4-6) |
