# Supplementary material for: Enhancing Team Strategies and Tools to Enhance Performance and Patient Safety Performance Through Medical Movies, Massive Open Online Courses, and 3D Virtual Simulation–Based Interprofessional Education: Mixed Methods Double-Blind Quasi-Experimental Study
Source: J Med Internet Res. 2025 Sep 8;27:e67001. doi: 10.2196/67001 (PMC12455160; doi:10.2196/67001)
Supplement: Multimedia Appendix 5 [file jmir_v27i1e67001_app5.docx]

| **Factors** | **Arm** | | | ***P* value** | | | |
| --- | --- | --- | --- | --- | --- | --- | --- |
|  |  |  |  | **Overall^a^** | **Pairwise comparisons^b^** | | |
|  | **A (n=29)** | **B (n=29)** | **C (n=29)** |  | **B vs A** | **C vs A** | **C vs B** |
| **Overall** |  |  |  |  |  |  |  |
| Mean (SD) | 2.04 (0.50) | 2.75 (0.75) | 2.88 (0.68) | - |  |  |  |
| Median (IQR) | 1.99 (1.63-2.29) | 2.75 (2.26-3.29) | 2.77 (2.37-3.32) | <.001^e^ | <.001^e^ | <.001^e^ | .594 |
| **Team Structure** |  |  |  |  |  |  |  |
| Mean (SD) | 1.54 (0.70) | 2.92 (1.10) | 3.35 (1.16) | - |  |  |  |
| Median (IQR) | 1.33 (1.00-1.88) | 3.29 (2.00-3.75) | 3.33 (2.50-4.50) | <.001^e^ | <.001^e^ | <.001^e^ | .160 |
| **Communication** |  |  |  |  |  |  |  |
| Mean (SD) | 2.21 (0.73) | 2.77 (0.91) | 2.97 (0.87) | - |  |  |  |
| Median (IQR) | 2.25 (1.75-2.50) | 2.75 (2.00-3.50) | 2.88 (2.25-3.75) | <.001^e^ | <.001^e^ | <.001^e^ | .424 |
| **Leadership** |  |  |  |  |  |  |  |
| Mean (SD) | 2.18 (0.64) | 2.88 (0.92) | 2.95 (0.81) | - |  |  |  |
| Median (IQR) | 2.00 (1.73-2.60) | 3.00 (2.00-3.60) | 3.00 (2.40-3.45) | <.001^e^ | <.001^e^ | <.001^e^ | .999 |
| **Situation Monitoring** |  |  |  |  |  |  |  |
| Mean (SD) | 2.60 (0.70) | 2.99 (0.64) | 2.90 (0.84) | - |  |  |  |
| Median (IQR) | 2.50 (2.20-3.00) | 3.00 (2.55-3.45) | 2.80 (2.50-3.40) | .003^d^ | .001^d^ | .027^c^ | .487 |
| **Mutual Support** |  |  |  |  |  |  |  |
| Mean (SD) | 1.67 (0.69) | 2.20 (1.01) | 2.22 (0.95) | - |  |  |  |
| Median (IQR) | 1.50 (1.25-2.00) | 2.00 (1.25-3.00) | 2.13 (1.25-2.75) | .002^d^ | .006^d^ | .002^d^ | >.999 |
|  | | | | |  |  |  |

Statistical tests: ^a^Kruskal-Wallis H test, ^b^Dunn's post hoc test

^c^*P*<.05, ^d^*P*<.01, ^e^*P*<.001
