# Supplementary material for: Enhancing Team Strategies and Tools to Enhance Performance and Patient Safety Performance Through Medical Movies, Massive Open Online Courses, and 3D Virtual Simulation–Based Interprofessional Education: Mixed Methods Double-Blind Quasi-Experimental Study
Source: J Med Internet Res. 2025 Sep 8;27:e67001. doi: 10.2196/67001 (PMC12455160; doi:10.2196/67001)
Supplement: Multimedia Appendix 6 [file jmir_v27i1e67001_app6.docx]

| **Rater Professions** | Pre-final assessment | | Post-final assessment | |
| --- | --- | --- | --- | --- |
|  | Percent agreement | Weighted Kappa | Percent agreement | Weighted Kappa |
| Doctor | 88.93% | 0.50 | 88.82% | 0.49 |
| Airway Nurse | 91.19% | 0.58 | 90.24% | 0.59 |
| Circulation Nurse | 93.45% | 0.71 | 93.87% | 0.76 |
| Pharmacist | 85.49% | 0.55 | 87.87% | 0.63 |
| Medical Technologist | 99.24% | 0.97 | 99.31% | 0.98 |
| Radiological Technologist | 95.38% | 0.77 | 96.06% | 0.84 |
