# Supplementary material for: Enhancing Team Strategies and Tools to Enhance Performance and Patient Safety Performance Through Medical Movies, Massive Open Online Courses, and 3D Virtual Simulation–Based Interprofessional Education: Mixed Methods Double-Blind Quasi-Experimental Study
Source: J Med Internet Res. 2025 Sep 8;27:e67001. doi: 10.2196/67001 (PMC12455160; doi:10.2196/67001)
Supplement: Multimedia Appendix 7 [file jmir_v27i1e67001_app7.docx]

| Themes based on Kirkpatrick’s model | Subthemes | Quotations |
| --- | --- | --- |
| Level 1 (Reaction) | | |
| Satisfaction | - Team  - Critical thinking  - MOOC  - Movie  - Game  - Connection between teaching media  - Feelings  - Debrief | Arm A  “I like playing this game. I feel like it was fun, but at first it might be a little stressful. I'm more worried because it's new. I am now in my 4th year but have never had an internship before. This game simulates *(real situations)*. Assume we meet real patients for the first time *(without hands-on practice)*. Our profession depends on the lives of the patients. The fact that we were able to prepare our minds beforehand by playing *(this game)* felt like it was very beneficial. And it's really good.” (A3-MT)  Arm B  “I prefer playing games as well, but I believe that to truly benefit, we need to have a debriefing session like this after playing. It might also be necessary to study a MOOC or learn some specific content before engaging in the game. If we just play the game without discussing it afterward, we might not gain much from the experience. However, if we combine gaming with post-game discussions, it becomes more meaningful. This approach also allows us to meet and get to know our friends better.” (B2-MT)  “When I saw the movie I thought it was very good. A hypothetical situation was created and we saw the roles of each profession and what their duties were. As a Pharmacy student, I pondered how we could contribute more effectively to this kind of teamwork. The MOOC provided concise briefings on each topic, and the video clips were short enough to keep them engaging and not tedious. I enjoyed the film; it was educational and offered a glimpse into real-life situations. In the area of ​​pharmacy, what part of this teamwork can we enhance? The MOOC is a short brief in each topic. The video clip is not too long, making it not boring. I like movies, I get to learn and see real situations.” (B4-P)  “In the movie, there is a scene where the doctor had a doubt about the patient's history of being really drunk or not. It delves into differentiating between structural and non-structural heart diseases. As someone with clinical training, these concepts are familiar to me. There are branches that separate it into structural, (or) no structural heart disease. As someone who has studied in the clinic, you have those stems in your head. *(So, I)* realized that the real event is like this. So, I asked about the patient history again by myself to confirm *and* diagnose again before proceeding with further treatment. Seeing that point in the movie is great.” (B4-D)  “The importance of interprofessional collaboration was clearly depicted in the scene where the doctor had to choose which patient to treat or refer first. In this scene, the doctor makes decisions based more on his emotions, lacking understanding of his team's feelings. This is evident when he speaks to his father, who asks if he knows how the team feels. The doctor admits his ignorance, attributing it to not observing or caring enough about the team, likely due to the stress and pressure at the time. In contrast, the MOOC provided a more theoretical viewpoint on how different professional steps interrelate, and its inclusion of movie scenes enhanced clarity. The issue of interprofessional assistance was evident in the scene where the doctor had to decide which patient to send for treatment or referral first. This was the scene where the doctor used his emotions more in making his decisions, and still did not understand the feelings of the team members much. Like the scene where he called his father, and then the doctor's father said that do you know how the people on the team are feeling right now? The doctor replied that how would he know? because the doctor hadn't noticed or paid much attention to the team. Perhaps, this was due to stress and pressure at that time. And from the MOOC, I think there was more of a theoretical perspective on how each step of work in each profession was related to each other. Also, in the MOOC, it was good that there were scenes cut from the movie, making it more clear.” (B5-RT)  Arm C  “It helps a lot with critical thinking. Everyone needs foundational knowledge to think or analyze effectively. Watching the MOOC offers this foundation, such as the ISBAR principles. Learning this made me realize what needs to be addressed, thereby enhancing my analytical thinking. Everyone who critically thinks or analyzes may need to have a basic knowledge. Watching the MOOC provides basic academic knowledge such as ISBAR principles. When you learn them, you know that we have to work on this issue. It affects critical thinking.” (C1-P)  “VR is more fun and realistic. If it were developed to be better, not heavy and able to play smoothly, it would be great. It's like we really went in and did it. This was fun. However, if you play for a long time, it's torturing. If your computer can only play games and you did not really try it out. It's like playing a normal computer game. And I think that the equipment placement in the game is too close together, making it difficult to press to select various equipment. (C1-N)  “I got a lot of critical thinking. I got a lot and felt that this is just one scenario. I feel that it creates a learning process similar to being in a real situation. And managing a real patient. When we enter the game, we are like taking that role so that we can think and decide and then change the decision. So it made us critically think and analyze. In each choice that we make, how does it affect the treatment of the patient? And it really has an impact. In the beginning of playing this game, I was not skilled. The thought process here may be disturbed because I was worried about using equipment. Unfamiliarity made us unable to focus on the thought process. But when I was proficient with the game, I feel like this game can create this very well.” (C2-P)  “The movie shows the picture more clearly. And it is something that I recall when thinking and analyzing systematically for an intervention or managing something or communicating something. I think of a movie as a basis more than a MOOC. The MOOC has good content but it is complicated. So, for each time of study, just listen and not understand it all. I must digest the information first. As for the movie, I watch it and understand it.” (C5-D) |
| Engagement | - Team  - Critical thinking  - MOOC  - Movie  - Game  - Comments  - Connection between teaching media  - Feelings | Arm A  “As for emergency vehicles, you have to get the contents inside by clicking it a little at a time, pressing down. You can't click to select to open that compartment. If possible, I want to be able to press that compartment and open only that comparment. No need to press down one by one.” (A3-N)  “Medical technology student didn’t get a chance to communicate much. There was only sending labs, receiving requests, informing results, and telling critical values. So, I didn't really communicate with other people much. I just had a little chat with the doctor.” (A4-MT)  Arm B  “Well, for me, I didn't have that much to do. That is, if we assume that we looked at the patient’s history, asked the patient questions, talked to the doctor, chose the medicine, and then the medicine was delivered. That was all, almost nothing else to do. That is, I sat and waited for quite some time. When it finished quickly, delivered and chose the medicine quickly, there were time left. Then, I sat and watched CCTV cameras to see what the doctor did. And then sat and watched until the end.” (B2-P)  “It may not be direct, but from observing teammates, in emergencies there will be unexpected events where the tube cannot be inserted and there is a broken ventilator in the game. It's an emergency. If it were real life, it would be the life of a patient. The fact that we can think critically and adjust according to the situation there is a skill that will help the patient survive in that situation. So, I thought it was great to play the game first. I also practiced critical thinking.” (B4-N)  “I learned to use ISBAR communication and call back a lot. Personally, when listening to it in the MOOC, I just felt so-so and questioned that it was really that important. At first, I thought that it was a waste of time to say the patient's name and HN every time. But when I actually did it, it didn't waste that much time and it was a recheck for myself and made sure that it was a right patient, really diagnosed this person so it didn’t cause any confusion. I think you need to practice speaking. Playing games like this about once or twice makes you start to absorb the words, but if you haven't encountered a real situation, haven't practiced, you think it's not important, just study and do not really pay attention to it.” (B4-D)  “Perhaps, add more situations because this one was used for both practice and the real play, same patient, same situation. You can add variety to it, so we will be able to play in many situations.” (B5-MT)  Arm C  “Thinking like what others have said, playing VR helps promote critical thinking skills and teamwork. We were in a semi-real situation. We got to play together, not just I playing alone with a computer system that automatically responded. But I got to play with other professions at the same time and have reactions. And the format in the game made it really tangible, such as clicking to see a real prescription, picking up real medicine. The sequence of steps was similar to a real situation where we really have to work like this.” (C1-P)  “I agree about the equipment as well. Although I was not being stressed at the beginning, playing for a while was stressful and tiring because the equipment was too heavy. As for the game, if we're talking about just the radiographic part, I think it might be too little because there is only a chest X-ray. Other things may be added.” (C1-RT)  “As for the lab, it's in the movie. You can see that the hospital in the movie doesn't have complete equipment. This makes it necessary to go back to using manual methods or standard methods, which is like increasing the learning process. In fact, we cannot rely on technology 100 percent. And there still needs to be some critical thinking about how we apply it to be able to examine and treat the patient.” (C2-MT)  “The advantage of the movie over the MOOC is that in the movie, we can clearly see the facial expressions of the actors. We can detect how each character feels and how they interact. The most obvious thing is the Medical technology student. It seems like the movie emphasized by clearly panning the camera in many scenes showing dissatisfaction, which is subjective for each character, while in MOOC, there may be critical thinking. But it may not capture everything as much as watching the entire movie, which is more obvious.” (C5-D) |
| Relevance | - Team  - Critical thinking  - MOOC  - Movie  - Game  - Comments  - Connection between teaching media  - Relevant knowledge/skills | Arm A  “I think the basis of the game is designed quite well. But the different thing is that VR exists. I thought that using VR would make it more smooth. This is the first point. The second point is that patients, like today, *(I)* thought that for some steps, it they were done more slowly, the patient may already be dead. But in the computer, it doesn't reflect this point. If we add a little more pressure here, it can be a more difficult level. It should be good and should help a lot with the practicet.” (A2-D)  “In the game, it's like just pressing. Suppose we take an X-ray, we will get an image. In fact, there must be post-processing. If we could add this level, it would be great.” (A2-RT)  “Because I had actually trained at a hospital, the game made it less than what I actually had experienced. I felt like there was nothing different from what I saw in real life. In the game, there was additional power *(for the character)*. I didn't press it at all because it's a game and you had to keep on pressing and pressing. But in reality, I would already speak out. The game is different…” (A4-P)  Arm B  “Regarding ISBAR communication, I think it can be used. From my internship, I have already used ISBAR, which is to check first whether the patient is correct or not. How is the situation? The things to be checked with the Request Order match or not. Most of them are already in use. So I feel like this is a lesson that can really be applied.” (B1-RT)  “For critical thinking skills in the game, it feels less than normal. For my profession, we are concerned with techniques for using tools. But in the game,  it's like there were not much options to press. For example, when adjusting the contrast for X-ray images, we have to adjust several things in reality, but we could just press and get the X-ray images in the game. Press the button immediately and it's finished. So, I don’t get much of anything here. It's like it's already fixed. So, it seems that our work was much less than that of other professions. I have a lot of free time, so it seems like I haven't really analyzed much. At the most, I just looked at the request to see if the doctor's order was correct or not, whether the order met the patient's condition, and just confirmed this. Otherwise, I felt that I got to think less than usual” (B2-RT)  “When I actually came to play the game, I have seen the importance of drug allergy, which is a big part in preventing repeated drug allergies, and analyzed together with the patient's risk factors. For example, this patient had COPD and had a history of being admitted to the ICU. We have to think about what infection might be the cause so that we can give empirical therapy to cover that infection. I have thought, analyzed and applied all of my knowledge to really manage the patient.” (B4-P)  Arm C  “I saw a more clear picture. I've never had an internship or seen what real work is like. When I watched it, I saw the importance of every professional role. It also made me aware of my own role. For example, the ISBAR principle, calling back, calling to report critical values. Maybe we are just shy, not daring to speak up, not arguing, not warning about things that he might do wrong and affect the patient. So, I think it makes us see the picture and realize patients is our ultimate goal.” (C1-MT)  “I agree with you guys. Watching the movie first, then watching a MOOC, and playing VR is like a teaching curriculum. I think it's similar, maybe better than the current teaching curriculum. The movie gives an overview of what our roles will be like. As soon as I saw this I want to learn theory and want to know this so that we can use it. At the university now, we study theory first. Next is internships or VR play, like job training, vocational training, hospital internships. I feel like all three, if possible, don't want to choose just one. And the sequence of this presentation method is that the research participants watch the movie first, then study the MOOC and then come play for real. This sequence is very good for me.” (C1-P)  “Give the same 70% because for actual work, I think there's more things to do for nurses than just what we did in the game. It's not just dealing with doctors or rooms. We have to deal with other parties such as social workers, take care of sick relatives, and take care of sick people. In the game, we communicated with only the doctor. But in real life, it's not just doctors. If, in the game,  there are additions besides the fact that the patient's relatives took pictures of the patient, there should be more to it than that, such as expenses, economic status. Does the patient have any other problems? It may be another form of communication other than this.” (C4-N)  “The downside of the game is that it doesn't show the player's facial expressions or emotions, which happen and are clearly seen in real life or movies. In the game, it doesn't clearly show them. So, when playing the game, we didn’t think about it. The only thing that comes to mind was the tone of voice when talking. The most I saw was when I turned to look at my friends playing. When we played using VR, we couldn’t see our friends anymore. When playing on the computer, I turned to my friend and saw that he/she was stressed. I saw his/her actions reflecting that emotion. So, I realized that when playing games, there is no this element.” (C5-D) |
| Level 2 (Learning) | | |
| Knowledge/ Skills | - Team  - Critical thinking  - MOOC  - Movie  - Game  - Comments  - Connection between teaching media  - Patient treatment | Arm A  “Perhaps, add some rejection of specimens, some inappropriate delivery. *In the game, the specimen* was sent, then we could automate and check almost every *specimen*. So, I thought that there should be some rejection. Let us practice rejecting specimens. Or maybe for the automatic machine *(in the game)*, it shouldn’t just putting in a blood vessel. There should be a bit of processing of the specimen before entering the machine. Let us learn a little more.” (A4-MT)  “For the role of radiological technologist, we must think about the safety of the patients and ourselves, such as cleanliness, i.e., cleaning tools” (A5-RT)  “I practiced taking the patient's history, including the symptoms, and thinking about what type of disease to diagnose and what investigations to send.” (A5-D)  “It helped. We communicated *(with other professions)*. If there was any problem, we always asked each other. I felt more courageous to ask. On the contrary, when working in the building, I do not dare to ask. So, there is a difference here.” (A5-N)  “I think it helps with critical thinking. There were certain moments in the game where we must educate, and helped the doctor choose the right medicine for the patient. As the patient's lab values ​​were critically high, we should find medicine to treat the symptoms.” (A1-P)  Arm B  “Taking care of patients requires many professions to take care of them, doctors, nurses, pharmacists, radiation technicians. Everyone is involved in taking care of them. Therefore, everyone must participate in a lot of communication. And to take the best care of patients is to communicate well, having the least mistakes. MOOC and movies help us see the importance of how we need to communicate, including ISBAR. As clearly seen, round 1 and round 2 were very different. Perhaps, it was because the teacher came to give us a summary and we reflected among ourselves. Maybe it was because the second time, the game had a pattern, it was noticeably better.” (B1-D)  “The movie shows how to give medicine to a patient. In the role of pharmacist, besides providing medicine to treat disease, risk factors for the patient must also be taken into account, such as conditions in which the patient may bleed. From taking warfarin  *(a name of the medicine)* in the movie, we must check as well and plan for more accurate medication administration.” (B1-P)  “And *(I)* learned to observe the emotions and feelings of teammates to see if they were ready to receive this message at that time or not, or having an uncomfortable mental state. This will cause the reception of the message to be incomplete or inaccurate, causing misunderstandings. This point must be noted as well. The MOOC, like my friend said, is a short clip with keywords. Just listen and listen, and these keywords just stick in my head. And while playing the game, these keywords came up that there were something like this. Although I can't remember everything, it helped a bit.” (B4-D)  “In the movie, I saw how everyone in the team worked and how they helped and depended on each other. It can be applied in the future when I graduate to work. It may not be an emergency room, maybe some other wards. But I also have to work with everyone on the team.” (B5-N)  Arm C  “The point of critical thinking in VR was clear because there was an example of a patient to be seen and managed by ourselves. In fact, I think it's OK. I had to arrange the order in which we looked at the patients according to what I've learned about in theory. And communication matters. Make me more courageous to ask questions from other professions. Dare to admit that I know little, don't know enough, or better to ask someone else. It helps a lot with this. If I haven't played this one yet, I might not dare to ask because I thought that whether others would think that I was the one who mainly had to manage the patients, being the lead in that situation, and I still didn’t know. I didn't know if others would be confident in me or not or what others would think. But after learning using VR, it gave me a better idea of being a team in helping manage patients, and dare to ask others who have more detailed or accurate knowledge than me” (C1-D)  “This helps me because it's like there are graphics displayed in the movie, showing thought processes of each profession. For example, in a scene where a doctor had to diagnose a disease, there was a pop-up saying which diseases there were that we needed to think about and then carefully considered the possibility. Then, cut the choices one by one. This one helps the thought process in diagnosing disease. What do we have to think about? Then, when finished, we cut out each choice one at a time. It will help develop the thinking process here.” (C2-D)  “*(I)* learned from the movie. *(I)* tried to analyze the scene as well *(by looking at)* the kind of effects facial expressions, and how people use the voices have. I felt that it was very good that when I was studying with MOOC, the instructor used scenes from the movie to explain the theory, making it possible to see that an event like this consistent with the theory. It's considered OK.” (C3-MT)  “*(I)* think that playing games helps practice more teamwork. Normally, on the ward, we only communicate with fellow nurses. But when playing the game, there was a group call at first. We practiced team communication and talked to the doctor about lab results. Practice more teamwork.” (C5-N) |
| Attitude | - Team  - MOOC  - Movie  - Game | Arm A  “If, in real life, communication is not understood, it will go wrong in the other direction. Work will be delayed and inefficient.” (A1-N)  Arm B  “Like I said, what I don't like about the game is that when we made mistakes, we couldn’t fix them. But actually, in real life, often making mistakes isn't a good thing. It's like a game helps us to think before we do something. In this way, it also helps to remind us because if we frequently make mistakes, not only delay will be caused, but also there will be a waste of resources.” (B2-N)  “When I saw the movie, I really saw the importance of communication and teamwork. In the movie, *(the characters)* must talk to each other, making us see the importance of communication to make a team truly a team, to know what is happening.” (B4-D)    “About the voice tones used in the movie, in the beginning *(of the movie)*, there was a new nurse, I saw that the nurse used the tone of voice when trying to communicate with the patient. That is, speak clearly. Therefore, I think that the tone of voice plays an important role in communicating to the team members to understand our intentions.” (B4-P)  Arm C  “What I thought at first was that, in the hospital, the doctor probably made every decision regarding the patient. But the movie changes that mindset. In the movie, it's a doctor who just graduated and was working to compensate for the scholarship he received. There were many things that the doctor still didn’t know. The doctor was not yet an expert and still didn’t know the environment of the new hospital. But nurses had been there longer. Nurses and other professions were consulted in many ways in matters related to the patient. Maybe the doctor needs to know. The doctor is the person who should know. But, in the movie, he still didn’t know. He asked others for the highest benefit of the patient. This changes the mindset of working as a team. Well, at first I thought it was the doctor who mainly made all decisions about the patient.” (C1-D)  “And then there is the issue of relationships among the team members. It will affect the efficiency of the teamwork. It is clear in the movie that in the beginning the team's relationship wasn’t on good terms. There were many problems in communication and it created burdens in terms of the mental health of the team. Therefore, having good team relationships will help increase efficiency in working as a team.” (C2-P)  “Give 60% *(of confidence level)* to communication. Communication can be used, but nurses communicated with only a few people because there were only a radiological technologist, nurses and a doctor *(in the game)*. But in real life, there may be more communication. I may talk to a medical technology student and a pharmacist for more details on specimens or medication administration.” (C3-N) |
| Confidence | - Team  - Critical thinking  - MOOC  - Movie  - Game  - Comments  - Connection between teaching media  - Patient treatment  - Level of confidence | Arm A  “About wearing protective equipment, I am more confident because we put it on many times. When I put it up, I still didn't know where to put it first. I only knew the bottom-to-top principle and saw some *(information)* in the clip. But *(the information)* is not the same in each clip that I have watched. When I came to do this, I feel like if I really need to wear it, I might be able to wear it. I knew some guidelines.” (A3-N)  “About 30% *(of confidence level)*, *(in the game)* it was that the patient having clear symptoms. So, it could be easily diagnosed. But in reality, it may be more difficult and you may have problems communicating with others.” (A4-D)  “The game did not require speaking. It might just be said that we had the equipment ready. Taking images was done as usual. It's not compulsory to talk about any other protocol. So, I think that it doesn't help much with communication in the work of radiological technologists. *(I)* give it about 40% *(of confidence level)*.” (A5-RT)  “About 60% *(of confidence level)*. From what I played, normally Medical technology students are only in the lab. Just now a doctor called and asked about the tube, so I was a little excited. It was good to practice communicating. He asked and I responded. It was fun and exciting too.” (A5-MT)  Arm B  “The skills learned are the same as my friends just said. I think the important *(skill)* is communications because it's like we have to work together. But if we can’t communicate and don’t understand each other, it might not go smoothly. It has to be communicated to understand and be considerate of each other at the same time. So, I felt like studying with the game and talking within the game seems to help a lot in this matter. Then, I went back and thought about it with myself to prepare for the improvement in the next round.” (B2-MT)  “*(I)* give three *(for my confidence)* as well. During the game practice, the doctor's request came in incorrectly. I was brave to point it out because I have seen MOOC that we should be the ones to recheck. *(I have)* confidence there. Patient identification is important as well.” (B3-RT)  “It is a matter of calling back to avoid mistakes while speaking verbally. For example, when the doctor issues an order and we phone back to repeat it, as well as when we administer medication to the patient. It will be a recurrent process to lessen our own mistakes. (B4-N)  It was mostly a matter of communication because we were not in the situation that much. We were outside.  It was a matter of communication where we must clearly communicate, *(inform)* the lab results and call back so that we could be confident that we produced accurate results.” (B4-MT)  “*(My)* confidence after playing the game remains the same 3-4. I saw the progress from the first, and the second time. It was a progress where I already knew mistakes from the first time, and I wouldn’t forget for the second time. I feel that, in real life, if I have ever encountered something similar like this before, some cases similar to this, I would know what to tell the nurses, pharmacists, etc. I think if I had known a little about that before, that I knew a little bit before actually seeing the patient, it would be better and make me more confident” (B5-D)  Arm C  “Regarding managing patients, shifting shifts, and communicating in a more orderly manner. Because, in the past, when I sent the shift, I still didn't get a clear picture. I just listened to the seniors and communicated all the points. That was enough. When I knew the theory from the MOOC or the movie, I could see more of the core structure and be able to understand more of what needed to be said. So, I could think ahead about what I need to know, what I must say to others in order to be complete and give the patient maximum benefits” (C1-D)  “*(I)* give 40% *(of confidence level)* because real patient information cannot be found like the one in the movie or the MOOC. Instantly using *(what I learned)* would take a lot more knowledge to analyze situations or provide basic patient care.” (C3-N)  “*(I)* give 50% *(of confidence level)* to both because both the movie and the MOOC taught basic thinking methods. I have had a lecture but haven't had a lab yet. I haven't actually practiced it yet. I still lack experience on the actual work. Will I be able to do it like him? Or if there are other events happening in the real work situation, can I apply this knowledge to that situation? I must continue practicing” (C4-MT)  “*(I give)* 70% *(of confidence level)* for the collaboration, which was clearly shown in the simulation. However, I deduct 30% *(of confidence level)* due to the diversity of situations. If it were a different scenario, there might be different guidelines for non-technical skills. I am not so confident.” (C5-RT) |
| Level 3 (Behaviors) | | |
| Effects of game practice on participant behavior | - Team  - Game  - Self review | Arm A  “The sequence of things to be done with the patient has some effects. As in the beginning, the x-ray came in first, but we hadn’t inserted a tube to help the patient to breathe yet. If it was a real situation, the patient would be in a bad shape. However, after the second game, I learned more and adjusted to have the nurse look at inserting the tube first, then finish drawing blood before taking the X-ray.” (A2-D)  “I think it has a lot of impact because, in the first round, as my friend said, we still weren’t quite sure what we needed to do next and we had a concern as it was very new. We just met for the first time since it was just watching clips, previously. However, after the second round, we knew that we were going to encounter this. Anxiety decreased and we knew what to do next.” (A3-MT)  “Played 2 rounds and I felt the same. *(After)* playing the second round, I didn't feel how much better it was than the first round. I could do it normally. The case was the same as before and I already understood the protocol. Communication was normally done. But I didn’t need to communicate much. It seems like there hasn't been much improvement for this matter.” (A5-RT)  Arm B  “Regarding learning to work as a team, for the movie and the MOOC, I feel like just knowing that it's important. But I still don't have a picture of where I have to put myself. When it comes to playing VR, when I put myself in the simulation, although I already looked at it *(i.e., the movie and the MOOC)*, knew it's important, and knew what's there, I still forgot. I didn’t do what I've learned. I feel that experimenting, debriefing, or reflecting after playing for the first time is quite effective. I saw a picture of what I lacked, what I needed to adjust, what shortcomings I had in order to improve myself. When I came to play round 2, I felt that errors hardly occurred at all. So, it is clearly seen that being in a simulated situation gives me a better picture of teamwork, particularly, in the matter of communication.” (B1-RT)  “It makes me remember more. And it makes me more accustomed to it. Because it's like when playing for the first time, I couldn't do anything right. I didn't know how to use this one. What should I choose and how? Therefore, the first round took a very long time. But once I started playing, I started to get used to it. And then after playing the first round, I sat and thought about where I did wrong last time. If I were to play the game next time, what would I adjust?...” (B2-P)  “Yes, because, during rehearsal, it was the same scenario, I already knew what the ending would be like. I didn't think about it that much again.” (B4-D)  Arm C  “When playing for the first time, I forgot some steps. After playing and at the end of the game, I just realized that I could do more. It was important. As I kept playing over and over, I learned from mistakes and did better. I feel like *(the game practice)* helped.” (C1-N)  “Playing the game over and over with the same scenario, same patient, same sequence, doesn't brought about more learning or analyzing. But if the patient's case is changed, I may learn more. But like today, learning repeatedly led to memorizing and not thinking or re-analyzing what I would do and what I should be careful about.” (C3-P)  “Become more familiar with the system and what to do. Where is each thing? Where do we click? There are probably various techniques that we learned each time. How do we pick up or release this thing in the most efficient way?” (C4-D) |
| Debriefing effects on participant behavior | - Satisfaction  - Relevance  - Knowledge/ Skills  - Attitude | Arm B  “*(Debriefing)* has some impacts. The first time that we had to try it ourselves, I didn't know where we needed to adjust. However, debriefing suggested what we should do, add, and fix. It made the next game better.” (B3-N)  “For many times, I knew it was wrong. But I just wanted to know what to do next, how to fix it, how to make it better. I feel that debriefing is very important in that part. I mean I used to think that I was just giving a summary. But when I saw the serious, detailed debriefing, the first time of the debrief, the instructors wrote down what I did and from what time until what time. It showed that the instructors were attentive to every action in the game. I feel that it really gives me the picture that this is taking too long a time. How can this be made better? I really see the importance of debriefing in that it tells me how I can be better. If you want to be better than this, what should you do?” (B5-D)  “Debriefing helps me know how I was in many situations and get suggestions that in the real life in the future, when working with interprofessional teams, I would not encounter this kind of comfort. I would face more pressure. It prepares us to enter into good work, which is considered OK.” (B4-MT)  Arm C  “*(Debriefing is)* very important. It allows me to see my own mistakes and sees which ones need to be improved. When we played, someone with more experience than us came and pointed out where it was wrong. If there is no debrief, even if we play 10 times, it is still not equal to someone who plays 2-3 times and has a debrief first and then comes to play afterwards because there are experts showing us the way. But if we continue to play by ourselves over and over, our basic knowledge may still not be equal to the experts.” (C1-P)  “Personally, I think it helps. Debriefing is like teachers pinpointing many points, and saying that at this point what the results would be if I did this. Sometimes, when I was playing *(the game)*, I didn’t think about it. After playing, I thought, but I still couldn’t figure it out or couldn’t see the picture. But the teachers helped by pointing out mistakes and told me how to improve or make it even better. Also, the point that I already did good and just kept doing what I did right.” (C2-N)  “I think it helps a lot too. As my friend said, it makes us see mistakes. But it's not just about making mistakes visible. But it also allows teammates to come and support our mistakes here. When we knew the mistakes of the team members, we brainstormed together and agreed on how to solve this problem.” (C5-RT) |
| ^a^Abbreviations denote the intervention arm, group number, and profession. Intervention arms included arms A, B, and C, and each arm consisted of five groups of participants. The following letters represent each profession: D for doctor, N for nurse, P for pharmacist, MT for medical technologist, and RT for radiological technologist. For instance, C5-N indicates an interview of a nurse in group 5 of the intervention arm C. | | |
